# INSIdE NANO: a systems biology framework to contextualize the mechanism-of-action of engineered nanomaterials

**DOI:** 10.1038/s41598-018-37411-y

**Published:** 2019-01-17

**Authors:** Angela Serra, Ivica Letunic, Vittorio Fortino, Richard D. Handy, Bengt Fadeel, Roberto Tagliaferri, Dario Greco

**Affiliations:** 1 NeuRoNe Lab, DISA-MIS, University of Salerno, Salerno, Italy; 2 Faculty of Medicine and Life Sciences, University of Tampere, Tampere, Finland; 3 Institute of Biosciences and Medical Technologies, University of Tampere, Tampere, Finland; 4 BioByte Solutions GmbH, Heidelberg, Germany; 5 Institute of Biotechnology, University of Helsinki, Helsinki, Finland; 6 Biomedicine Institute, University of Eastern Finland, Kuopio, Finland; 7 School of Biological and Marine Sciences, University of Plymouth, Plymouth, United Kingdom; 8 Institute of Environmental Medicine, Karolinska Institutet, Stockholm, Sweden

## Abstract

Engineered nanomaterials (ENMs) are widely present in our daily lives. Despite the efforts to characterize their mechanism of action in multiple species, their possible implications in human pathologies are still not fully understood. Here we performed an integrated analysis of the effects of ENMs on human health by contextualizing their transcriptional mechanism-of-action with respect to drugs, chemicals and diseases. We built a network of interactions of over 3,000 biological entities and developed a novel computational tool, INSIdE NANO, to infer new knowledge about ENM behavior. We highlight striking association of metal and metal-oxide nanoparticles and major neurodegenerative disorders. Our novel strategy opens possibilities to achieve fast and accurate read-across evaluation of ENMs and other chemicals based on their biosignatures.

## Introduction

**Figure 1:**
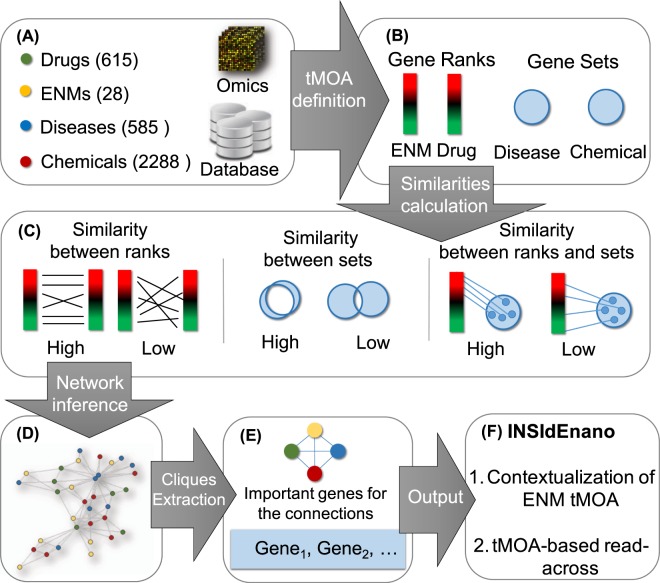
INSIdE NANO workflow. Transcriptomics data (ENMs (n = 28) and drugs (n = 615)) and precompiled lists of associated genes (Human Diseases (n = 585) and Chemicals (n = 2288)) were retrieved from multiple sources (**A**). tMOA signatures were derived for each phenotypic entity in form of gene ranks for ENMs and Drugs exposure and gene sets for human diseases and chemical exposures (**B**). tMOA based pariwise similarity were computed (**C**). Pairwise similarities were used to infer a weighted network of phenotypic entities (**D**). Cliques and their associated list of genes underlying the connections were identified (**E**). INSIdE NANO achieves contextualization of ENM tMOA and to perform tMOA-based read-across analysis (**F**).

**Figure 2:**
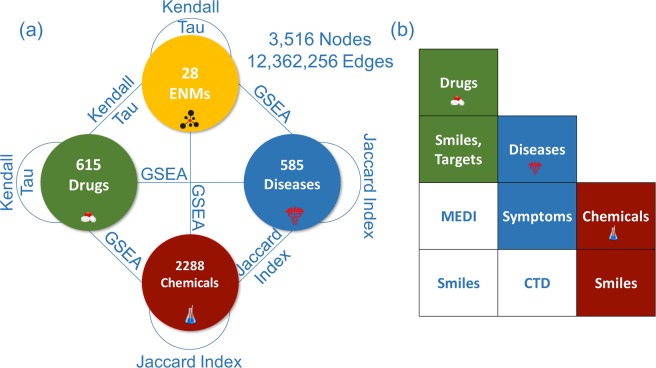
INSIdE NANO data and architecture. The phenotypic entities in the discovery data sets were integrated to perform ENMs contextualization. The INSIdE NANO network contains 28 ENMs, 615 drugs, 585 human diseases and 2288 chemicals connected by 12,362,256 edges. The weight on the edges are proportional to the strength of similarity between the entities. This similarity was computed by means of different metrics: the Kendall Tau distance was used to compute similarities between the ranked list of genes associate to the ENMs and drugs; the Jaccard Index was used to compute similarities between the sets of genes associated to Chemicals and Diseases; the Gene Sets Enrichment Analysis (GSEA) was used to compute similarities between the ranked list of genes associated to the ENMs and Drugs and the sets of genes associated to chemicals and diseases (**a**). Data sets used to validate the connections inferred in the INSIdE NANO network. The similarity between the entities based on the molecular alteration profiles were validated by comparing it with already computed similarity measures unrelated from the molecular alterations. Drugs similarities were compared with smiles and target based similarities. Diseases similarities based on symptom were computed, while chemicals similarities are computed using smiles. Drugs and diseases similarities were computed based on prescription information downloaded from the MEDI database. Drugs chemicals simililarities were based on smiles and disease chemicals similarities were download from the CTD database (**b**).

ENMs already pervade our everyday lives, being present in numerous consumer products, and new nanomaterials are being produced at an ever-increasing pace. However, despite considerable advances in the past decade, we are still far from a comprehensive understanding of the biological effects of the myriads of existing and emerging ENMs^1,2^. Global omics technologies may aid in characterizing the mechanism-of-action (MOA) of ENMs, opening new possibilities for next generation safety assessment based on systems biology approaches^3^. An emerging strategy in risk assessment of chemicals is read-across analysis, under the assumption that structurally similar compounds exert comparable biological effects. To date, only a few read-across analyses have been proposed for ENMs due to the limited possibility to computationally derive their physical-chemical properties, for their molecular size and complexity. Moreover, only marginal attempts have been made to integrate MOA signatures in read-across, even when evaluating structurally smaller and simpler compounds. However, the notion that any phenotypic perturbation produces a specific pattern of molecular alterations that can be used as its signature is well established, for instance, in studies of drug repositioning^4–6^. Based on the hypothesis that an effective drug should be able to counterbalance the perturbations caused by a disease, correlations between disease- and drug-associated gene expression signatures have been sought in attempts of repositioning drug molecules^7^. Interestingly, the biological effects of chemicals have not yet been exploited in a systematic relationship with the molecular signatures of human diseases, which in turn could add significant amount of information to the read-across evaluation. Here, we hypothesized that systematic analysis of transcriptional mechanism of action (tMOA) signatures could be used to contextualize or ‘position’ ENMs with respect to human diseases, drug treatments, and chemical exposures. This strategy could mitigate the current limitation of information available concerning ENMs effects. Moreover, knowledge on the molecular effects of ENMs could be also used to identify adverse outcome pathways that may lead to pathogenesis, or indeed tMOA of ENMs that facilitate their application as potential treatments. To allow for systematic contextualization of the effects of ENMs, we developed the computational tool INSIdE NANO (‘Integrated Network of Systems bIology Effects of NANOmaterials’, available at http://inano.biobyte.de, and briefly described in methods section). To this end, we derived, from the scientific literature or from the analysis of available transcriptomics data, specific tMOA signatures of a large set of human diseases (the full list is reported in Data S1), chemicals (Comparative Toxicogenomics Database - CTD^8^, the full list is reported in Data S2), FDA-approved drugs (Connectivity Map Database - Cmap^9^, the full list is reported in Data S3), and ENMs (NanoMiner^10^ - the full list is reported in Data S4). Gene expression data for ENMs exposure analyses were retrieved from NanoMiner, a public transcriptomics database encompassing *in vitro* transcriptomics profiles obtained in human cells or cell lines for a panel of ENMs. See Supplementary Materials and Fig. S1 for details on input data and preprocessing. We then computed the degree of similarity between all the pairs of biological entities present in this integrated data set based on the similarity of their tMOA signatures. In particular: (i) the Jaccard index was used to compute pairwise similarity between gene sets; (ii) the Kendall Tau distance was used to compute similarity between ranked lists of genes; (iii) the Gene Set Enrichment Analysis (GSEA) was used to compute similarities between ranked list of genes and gene sets. We then used this information to build a large network of 3,516 nodes (phenotypes) interconnected by 12,362,256 edges. The work-flow of the analysis, the database architecture and the data integration strategy are schematically shown in Figs 1 and 2 and described in details in the method section.

## Results

### Definition of the INSIdE NANO phenotypic network

We integrated tMOA signatures of four types of phenotypic entities (ENMs, drugs, human diseases and chemical substances), either derived from de novo transcriptomics data analysis or from scientific databases. We studied the patterns of similarity of these tMOA signatures, and used them to predict the biological effects of ENMs. We defined a list of associated genes for each phenotypic entity to be its tMOA signature. In our analysis, tMOA of ENMs and drugs are represented by ordered lists of genes ranked by their differential expression values. Furthermore, tMOA of chemicals and molecular alterations of diseases are represented by sets of associated genes retrieved from the Comparative Toxicogenomics Database (CTD) (Figs 1A and 2a and method section for a description of the input data). We hypothesized that the relatedness of each pair of perturbations (ENMs, drugs, chemicals and diseases) can be quantified as the degree of similarity between their specific tMOA patterns. Following data homogenisation (Fig. 1B,C, and method section for a description of the similarity measures), the integrated pairwise similarity matrix was used as an adjacency matrix to construct a weighted undirected interaction network, which we called INSIdE NANO, where the nodes are the phenotypes (ENMs, drugs, diseases and chemicals) and the tMOA similarities between them represent the edge weights. We also retained the information on the direction of the similarities (positive or negative), so that the edges in the network have a sign attribute indicating if the tMOA signatures of two nodes are concordant (the genes are altered in the same direction by both the perturbations) or discordant (the genes are altered in the opposite direction by the two perturbations). See Figs 1D and 2a and method section for a description of the network inference process.

### MOA signatures mirror chemically, biologically and clinically relevant patterns

One of the factors preventing omics technologies from being fully integrated in regulatory assessment of chemicals is the “noisy” nature of the MOA signatures usually derived from these high-content assays. We thus tested the hypothesis that our computational framework, inferring similarities between phenotypic entities from their tMOA signatures, can also highlight robust information that corresponds to either structurally driven (as implemented in currently established read-across methods) or clinically relevant patterns of similarity. To this end, we systematically computed pairwise similarity matrices between the sets of phenotypic entities present in our analysis and independent data sets concerning other relevant aspects unrelated from their molecular effects (Figs 1C and 2a). Next, we assessed the correlation between these similarity patterns and those derived from our integrative tMOA analysis (Table 1). See section method for more details. We indeed confirmed that our tMOA-based similarities significantly resembled those computed by considering independent characteristics, such as the 2D molecular structure of the drugs (Mantel’s test *P* < 0.01) and chemicals (Mantel’s test *P* < 1*E* − 05), respectively. In addition, our tMOA-derived relatedness of drugs could also successfully recapitulate their analogy based on known molecular targets (Mantel’s test *P* < 1*E* − 05). Interestingly, also structural similarities between drugs and chemicals were significantly similar to those computed from tMOA signatures, although derived from different data sources (Mantel’s test *P* < 1*E* − 04). Similarly, we observed substantial consistency of our disease-disease similarities based on patterns of molecular alteration with those calculated by taking into account the overlapping clinical symptoms (Mantel’s test *P* < 1*E* − 05). Furthermore, our inference was substantially coherent to the known drug-to-disease relationships based on the use of specific drugs to treat certain diseases in clinical practice (Mantel’s test *P* < 1*E* − 04) as well as known chemical-to-disease connections based on epidemiological causal evidence of the pathogenic effects of exposures (Mantel’s test *P* < 1*E* − 05). Taken together, these results strongly support that our strategy of data integration and homogenization is robust and allows highlighting meaningful relationships between phenotypic entities of different types.

**Table 1:** INSIdE NANO associations based on tMOA similarities.

INSIdE NANO (MOA)	Similarity By	Mantel’s Test P
Drugs - Drugs	chemical structures	1*E* − 02
Chemicals - Chemical	chemical structures	1*E* − 05
Drugs - Chemicals	chemical structures	1*E* − 04
Drugs - Drugs	molecular targets	1*E* − 05
Diseases - Diseases	symptoms	1*E* − 05
Drugs - Disease	use in clinical practice	1*E* − 05
Chemicals - Diseases	pathogenic exposures	1*E* − 04

The correlations (similarities) between certain types of biological entities (in rows) computed based on the transcriptional mechanism-of-action (tMOA) similarity were systematically compared to those calculated considering independent biochemical aspects. Mantel’s test P is reported, under the null hypothesis that two compared matrices are different.

### Extrapolation of phenotypic cliques reveals connections between ENMs and respiratory and dermal diseases

Graphs (or networks) can efficiently represent complex phenomena and they can be rapidly analyzed with ad hoc algorithms that consider the patterns of relatedness of their constituents. We hypothesized that degrees of tMOA-derived similarity between sets of phenotypes could be used as an indication of biological association. Specifically, we scanned INSIdE NANO in search of ‘clique’ subnetworks, i.e., quadruplet structures of heterogeneous nodes (a disease, a drug, a chemical and an ENM) completely interconnected by strong patterns of similarity or anti-similarity (Fig. 1E). More details on the search algorithm are reported in the method section and Fig. S2. We could validate our predictions related to the relative proximity and connectivity of phenotypic entities in our network against a set of known associations between diseases and drugs (Kolmogorov-Smirnov test, *P* < 0.002), based on drug use in clinical practice^11,12^, and between diseases and chemicals (Kolmogorov-Smirnov test, *P* < 0.001), based on literature analysis. Chemical-disease interaction data were retrieved from the CTD. Further, the list of heterogeneous cliques of size three and four was ranked to identify the most robust ones. Firstly, since lower thresholds in the clique search algorithm denotes higher connectivity strength between the nodes, only the cliques identified with a threshold lower or equal than 0.4 were selected. We then focused our analysis on the cliques including at least one known connection. A permutation test was executed (as described in the methods section) to asses the significance of the subset of cliques. Only the cliques with high connection strength, at least one known connection, and significant pvalue (*pvalue* < 0.05) where finally selected. We then focused on the possible involvement of ENMs in the most robust identified cliques and inferred connections between specific ENMs and several human diseases, including, for instance, conditions affecting the respiratory system and skin (Figs S3–S7). The latter observations are strongly corroborated by the well established notion in literature about the pulmonary and dermal effects of certain ENMs.

### Association of metal and metal oxide nanoparticles with neurodegenerative disorders

**Figure 3:**
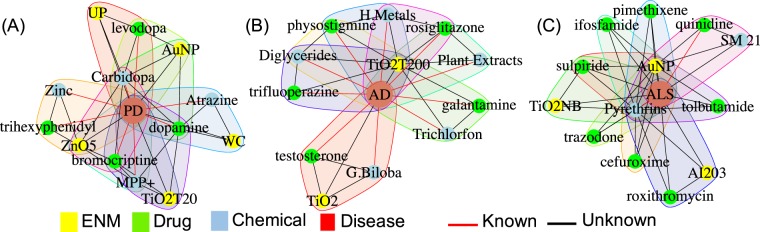
Significant association between ENM and neurodegenerative diseases. Relevant top-10 cliques including associations between ENM, chemicals and drugs MOA with Parkinson’s disease (**A**), Alzheimer’s disease (**B**), amyotrophic lateral sclerosis (**C**). Cliques including at least one known connection between disease-drug and disease-chemical were selected.

**Figure 4:**
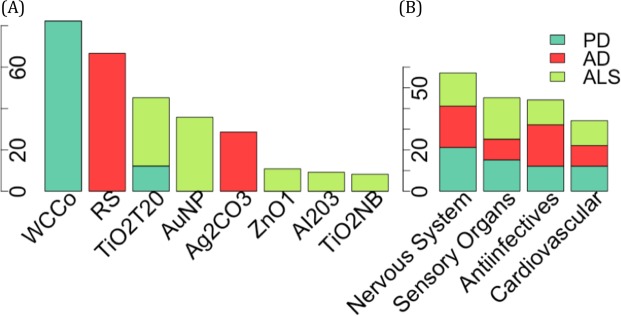
The cliques including at least one known connection between the disease-drug and disease-chemical were selected. The number of significant interactions between Parkinson disease (dark green), Alzheimer disease (red), and amyotrophic lateral sclerosis (light green) and each ENM (X-axis) are depicted as barplot (**A**). The drugs included in the significant cliques, categorized by the first level of their ATC code, are shown as bar plot (**B**).

Our systematic search of cliques highlighted a subset of intriguing tMOA similarity patterns related to three important neurodegenerative disorders, i.e., Parkinson’s disease (PD, Figs 3A, S8, Data S5), Alzheimer’s disease (AD, Figs 3B, S9, Data S5), and amyotrophic lateral sclerosis (ALS, Figs 3C, S10, Data S5). We focused on the most significant cliques where disease-drug and disease-chemical associations were already known and investigated the potential connections of ENMs in this context. Our analysis clearly pointed to an association between metal and metal oxide nanoparticles (NP), including tungsten carbide cobalt (WCCo), titanium dioxide (*TiO*_2_), zinc oxide (*ZnO*), and gold (*Au*), and neurodegenerative disorders (Fig. 4A). The neurotoxicity of metals, such as lead, mercury, aluminium, cadmium, and arsenic, is well known^13–15^. There is also some evidence for a relationship between inhaled particles, e.g., ultrafine particle exposures in ambient air or at the workplace (e.g., metal fumes) and neurotoxicity in humans^16–18^. We found *WCCo* NP to be strongly associated with PD (Figs 3A and S8), together with the neurotoxin 1-methyl-4-phenylpyridinium (MPP+), which is known to cause PD by destroying dopaminergic neurons in the brain and its prodrug 1-methyl-4-phenyl-1,2,3,6-tetrahydropyridine (MPTP). Moreover, the anti-PD drugs levodopa, dopamine and bromocriptine completed the PD-related cliques (Figs 3A and S8). *WCCo* NP are known to be cytotoxic and genotoxic, and astrocytes cultured *in vitro* were found to be the most sensitive in a study involving a range of mammalian cell models^19^. To the best of our knowledge, there are no *in vivo* studies on *WCCo* effects on the CNS. However, further investigation should address the possibility that *WCCo* NP may be especially harmful for the brain. The potential neurotoxicity of *TiO*_2_ NP has already been investigated both *in vitro* and *in vivo*^20,21^. *TiO*_2_ NP are easily translocated into the brain of exposed mice, either via the blood-brain barrier or the nose-brain path, but their elimination rate is limited, thus resulting in their accumulation and consequent damage of neurons and glial cells^22^. It is of interest to note that different *TiO*_2_ NP are identified in different cliques, suggesting that differences in material properties are associated with distinct disorders (Figs 3 and S8–S10). For instance, the *TiO*_2_ nanobelts (NB)^23^ were found to be associated with ALS, while spherical *TiO*_2_ NP (of different primary particle sizes) were associated with AD and PD. Diethylene-glycol coated *ZnO* NP, but no other types of *ZnO* NP integrated in INSIdE NANO, were significantly associated with both PD (Figs 3A and S8) and AD (Figs 3B and S9). In a previous *in vitro* study, a panel of nine *ZnO* NP were tested for their cytotoxicity potential using the Jurkat leukemic cell line^24^. Diethylene-glycol-ZnO was found to be the most cytotoxic of all the *ZnO* nanoparticles tested and also elicited the strongest transcriptomic response among the screened nanoparticles^21,24,25^. Interestingly, Xie *et al*. reported that repeated administration of *ZnO* NP elicited behavioral and electrophysiological improvements in a rat model of depression^26^. We also observed a significant association of *Au* NP with PD (Figs 3A and S8) and ALS (Figs 3C and S10), a devastating neurological disease characterized by the death of motor neurons. Interestingly, *Au* NP have been shown to induce oxidative stress and to reduce the activity of antioxidant enzymes in rat brain^27^. Moreover, exposure to *Au* NP decreased the levels of the neurotransmitters dopamine and serotonin. It is pertinent to note that gold is widely used for the treatment of rheumatoid arthritis (RA) and that neurotoxicity has been documented in patients with RA receiving oral or injectable gold^28,29^. Whether or not *Au* NP also elicit similar effects is unknown. Other elements retrieved in the context of the *Au* NP-ALS connections using the INSIdE NANO tool included quinidine and pyrethrin (Figs 3C and S10). Quinidine, in combination with dextromethorphan, is used to treat affective disorders in patients with ALS^30^. Pyrethrin, on the other hand, has insecticidal activity by targeting the nervous system of insects^31^. Taken together, these results suggest that INSIdE NANO does not indiscriminately group ENMs based on their core chemistry, and provides evidence for the importance of other physicochemical properties, including, in the case of *ZnO* NP, the surface coating and attendant rate of particle dissolution, and, in the case of *TiO*_2_ NP, the shape or aspect-ratio of the particles, as discussed above.

### Toxic effects of metal and metal oxide nanoparticles on the central nervous system *in vivo*

Clinical case studies that demonstrate an association between exposure to ENMs and neurodegenerative diseases in humans are currently missing. Inevitably, given the latency of these diseases it will be some decades before occupational health data becomes available from exposure monitoring in the workplace, or from traditional epidemiology in public health. However, the fundamental events in chemical toxicology that may lead to brain injury are known. Figure S11 shows the key events in the adverse outcome pathway (AOP) leading to human disease. The involvement of ENMs has been demonstrated *in vivo* for key events in the AOP at the molecular/biochemical, physiological and pathophysiological levels. The etiology of brain injury from ENMs includes oxidative stress, ionoregulatory disturbances, brain pathology, and changes in fish behavior, that can only be explained by neurological deficit. The *in vivo* studies (Table S1) mapped onto the AOP have been carefully selected to be brain-specific and not caused by secondary systemic hypoxia (e.g., from respiratory distress) than can indirectly lead to brain injury. Figure S11 shows ENM involvement in most of the steps of the AOP, suggesting that the INSIdE NANO predicted associations between metal nanoparticles and neurodegenerative disorders are recapitulated in an *in vivo* model. Our analysis also highlighted key genes, whose expression is altered by specific metal and metal oxide NP, potentially involved in mediating the pivotal steps in the pathogenesis of Parkinson’s disease (Table S2), Alzheimer’s disease (Table S3), and amyotrophic lateral sclerosis (Table S4). Taken together, our results not only are able to facilitate rapid prediction of possible implications of ENMs exposure in human pathogenesis, but provide also strong evidence for possible key molecular events mediating the ENMs effects.

### Potential application of INSIDE NANO for drug (re)positioning

Drug-drug and drug-disease tMOA-based similarity patterns inferred in INSIdE NANO significantly mirrored those derived from chemical and clinical evidence (Table 1), thus suggesting that INSIdE NANO could also serve as a discovery tool for drug positioning. Along this line, we observed that the drugs in the significant cliques involving neurodegenerative disorders are known to target the nervous system and sensory organs (Fig. 4B). In addition, anti-inflammatory molecules and drugs known to exert their therapeutic effect on the cardiovascular system were also retrieved in connection to neurodegenerative disorders (Fig. 4B). We recently described computational repositioning of many compounds acting on the cardiovascular system as neuroactive drugs, probably due to similar molecular structure and MOA, which often affects the stability of the membrane potential^6^. Based on these results, it is possible to argue that positioning of ENMs for biomedical applications is also conceivable, using INSIdE NANO.

## Discussion

In the post-genomic era, omics studies have been routinely used to address a plethora of biomedical questions and, consequently, enormous amount of omics data and omics-derived information are accumulating. Although the value of omics screenings has been recognized also in the field of chemical safety, to date the use of these technologies is mainly limited to the measurements of the primary molecular responses to drugs or chemicals. This information, in turn, is used to characterize the MOA during exposures and defining pathways of toxicity (PoT) that could serve as biological signatures. Given the increasing amount of data regarding the tMOA of drugs and chemicals, the next challenge appears to be the systematic integration of these exposure-specific biological signatures with the patterns of molecular alteration of human diseases. This could greatly help the positioning of chemicals and drugs as toxicants or therapeutics to a specific disease, and hence provide a valuable indication in terms of hazard assessment as well as drug development. However, lack of standardization in the computational strategies and algorithms used for deriving and comparing tMOA signatures has, until now, prevented omics data from being fully exploited in safety assessment. In this study, we assumed that comparisons of tMOA signatures could be used to find robust and meaningful relationships between different types of exposures and human diseases. Overall, our results demonstrate that this is indeed possible by integrating different types of data, including omics. Moreover, the rigorous validations of our novel data analysis and integration methods suggest that our computational framework could pave the way to a complete integration of omics technologies into regulatory read-across analysis. Read-across is rapidly becoming a strategic instrument to meet the increasing need to perform rapid assessment and labelling of many compounds, including ENMs^32,33^. This knowledge gap-filling strategy traditionally consists of defining groups of molecules with high structural similarity, under the assumption that they will also exert similar biological effects. Currently, read-across systems present several limitations. First, although otherwise envisaged, they are usually restricted to partial chemical spaces consisting of sets of compounds with relatively homogeneous applications/effects, limiting their applicability domains. In this context, the analysis of ENMs is hampered by the difficulties to computationally derive structural descriptors to be implemented in read-across systems, and hence only few studies limited to specific classes of ENMs have been proposed thus far^34–36^. Second, except for a few valuable attempts^37^, read-across mostly relies on grouping ENMs or chemicals based only on the similarity of their molecular structure, neglecting their MOA. Third, read-across systems so far work on specific endpoints of strict toxicological interest; and do not strive for the possibility to directly infer exposure-disease relationships which could also be used to position an exposure as drug^38^. The work presented here significantly addresses each of these limitations. In fact, we could successfully analyze, in the same property space, different types of drugs and chemicals, and ENMs. INSIdE NANO broadens the classical evaluation of chemical exposures, based on the structural properties of the compounds, to their primary tMOA. Doing so, we retrieved relevant information about ENMs and their effects by contextualizing their molecular behavior with respect to multiple phenotypic entities (ENMs vs chemicals, drugs and diseases). To the best of our knowledge, this is the first attempt to analyze the molecular effects of ENMs in the context of a larger space including other chemicals, drugs, and human diseases. Moreover, we demonstrated that, when accurately derived and interpreted, similarity patterns of omics-derived tMOA are able to recapitulate structural analogies of the compounds as well as clinically relevant relationships between diseases, drugs and diseases, and chemicals and diseases. Finally, our methods provide a systematic way to infer robust implications of exposures to human diseases, going beyond specific toxicology endpoints, which can be difficult to link to human pathogenesis. An important regulatory and ethical issue is the possibility to derive organism-level information from *in vitro* assays. Matching tMOA signatures of drugs tested *in vitro* with patterns of molecular alterations of patients has already proved valid in suggesting drug repositioning^7^. Moreover, we have recently demonstrated that a gene network-based analysis of omics data allows to highlight molecular pathways consistently altered by ENMs exposure *in vitro* and *in vivo*^39^. Along the same lines, here we integrated tMOA signatures derived *in vitro* (ENMs, drugs, and some chemicals) and *in vivo* (diseases and some chemicals). Importantly, the associations between ENMs and neurodegenerative disorders computationally predicted by INSIdE NANO are recapitulated in a whole body *in vivo* exposure model in fish, and also rodents. It should be noted that omics screening *in vitro* can be used to identify the tMOA associated with an exposure, which is the ensemble of the primary molecular alterations caused by that exposure. In this sense, *in vitro* experiments can be of great value in inferring pathways of toxicity. We acknowledge that the current lack of data concerning ENM MOA poses a challenge in respect of the potential of INSIdE NANO and future iterations of the tool will take into account new data as these become available. However, despite this potential limitation, we were already able to derive meaningful and statistically robust similarities between ENMs, drugs, chemicals, and human diseases. In conclusion, we have developed INSIdE NANO, a novel computational platform for the systematic contextualization of ENMs tMOA in relation to human diseases, drug treatments, and chemical exposures. Our analysis of the large integrated data set underlying INSIdE NANO has pointed towards novel associations of specific metal and metal oxide nanoparticles with neurodegenerative disorders, and underscores the utility of transcriptomics analysis *in vitro* for the prediction of possible *in vivo* effects of ENMs. These results suggest that epidemiological studies of the possible relationships between exposure to metal based nanoparticles and neurodegeneration are warranted to establish whether ENMs are a risk factor for such disorders.

## Methods

### Data integration

For each phenotypic entity, a list of associated genes is given. In particular, a set of genes is associated to each disease and chemical, while an ordered list of genes resulting from differential expression analysis is built for each drug and ENM in the data set. In order to construct a similarity network between the phenotypic entities all the pair-wise similarities between them were evaluated (Figs 1C and 2a).

#### Gene set *versus* gene set similarity

The Jaccard Index was used to compute the pair-wise similarity between gene sets (two diseases, two chemicals or a disease and a chemical). Given two sets A and B the Jaccard index is defined as: $J(A,B)=\frac{|A\cap B|}{|A\cup B|}$. This measure is 0 if the intersection between A and B is empty, while it is 1 if it contains exactly the same elements. For each chemical, two sets of genes were considered: those whose expression is up-regulated and those whose expression is down-regulated by the chemical exposure. For the down-regulated genes, the Jaccard Index was multiplied by −1 in order to take into account the effects on the genes.

#### Gene rank *versus* gene rank similarity

After importing the pre-processed NanoMiner and CMAP datasets in R, a contrast matrix for each dataset was constructed by using the limma package; only the subset of shared genes in both datasets was considered. For the NanoMiner dataset, the contrasts were defined to compare each sample exposed to an ENM against the controls. Likewise, for the CMAP data, contrast is defined considering each drug versus the untreated controls. Subsequently, the genes were ranked by using the following score $\pm logFC\cdot -\,log(Pval)$, resulting in ordered gene lists having the most up regulated genes on the top and the most down regulated genes in the bottom. The Kendall Tau Distance^40^ was then used to evaluate the similarity between ENMs, drugs and ENMs-drugs based on the ranked lists of genes. The Kendall Tau distance between two lists *T*_1_ and *T*_2_ is defined as follow:

1 $$\begin{array}{rcl}K({T}_{1},{T}_{2}) & = & |\{(i,j):i < j,(({T}_{1}(i) < {T}_{1}(i))\\  &  & \wedge \,({T}_{2}(i) > {T}_{2}(j)))\vee (({T}_{1}(i) > {T}_{1}(j))\\  &  & \wedge \,({T}_{2}(i) < {T}_{2}(j)))\}|\end{array}$$

where *T*_1_ and *T*_2_ are two ranked lists of genes. Their values range between 0 and *n*(*n* − 1), where n is the list length. A value of 0 means that elements in the list are in the same order; A value of *n*(*n* − 1) means that elements in the list are in the opposite order. Values were finally normalized to the range [−1; 1] where −1 corresponds to *n*(*n* − 1) and 1 corresponds to 0.

#### Gene rank *versus* gene set similarity

The Gene Set Enrichment Analysis (GSEA)^41^, based on the Kolmogorov-Smirnov test, was used to compute the pairwise similarity between an ENM and a disease, and an ENM and a chemical, a drug and a disease, and a drug and a chemical. The Kolmogorov-Smirnov test^42^ can be used to compare a sample with a reference probability distribution. The empirical distribution function *F*_*n*_ for *n iid* observations *X*_*i*_ is defined as ${F}_{n}(x)=\frac{1}{n}\,{\sum }_{i=1}^{n}\,I[\,-\,\infty ,x]({x}_{i})$ where $I[\,-\,\infty ,x]({X}_{i})$ is the indicator function defined on a set X that indicates the membership of an element to a subset *A* of *X*, having the value 1 for all elements of *A* and the value 0 for all elements of *X* not in *A*. The Kolmogorov-Smirnov statistic for a given cumulative distribution function *F*(*x*) is *D*_*n*_ = *sup*_*x*_|*F*_*n*_(*x*) − *F*(*x*)|. As in^43^, the Kolmogorov-Smirnov statistic was used without the absolute value in order to preserve the sign. This helps understanding if the genes in the sets are up or down-regulated.

### Phenotypic Network Inference

The pairwise similarity matrix was used as an adjacency matrix to construct a weighted undirected network where the nodes are the entities and the similarities between them represent the edge weights. Each similarity measure has a different range of values. To make them comparable, these values were scaled in the uniform range 0–1 by means of the cumulative function. Unlike the similarity value, the signs have not been altered, and then edges in the network have a sign that indicate if the correlation between a couple of nodes is positive or negative. The resulting network is completely connected. To reduce the number of nodes and analyze only strong connections, we used a ranking system to cut edges. For each vertex we ranked its neighbors basing on the similarity score; then we can query the network by setting a percentage of the top edges to select (e.g. first 10%, 20%, 30% of the rank). Since rankings are not symmetric, when we cut the ranked list we compute the mutual neighborhood of a node i defined as ${\mathscr{N}}(i)=\{j:(ran{k}_{i}(j)\le th)\wedge \,(ran{k}_{j}(i)\le th)\}$, where *rank*_*i*_(*j*) is the position of node j in the ranked list of nodes connected to i and *th* is the user defined threshold.

### Cliques Search

A graph or network is a mathematical abstraction that represents a set of objects (nodes) and their relationships (edges). Formally, a graph *G* is defined as the pair *G* = (*V*, *E*), where $V=v1,\ldots ,vn$ is the set of the nodes of the graph, and $E=e1,\ldots ,em$ is the set of the edges. Each edge in *E* is a connection between a pair of nodes (*x*, *y*) in *V*. If a relevant sorting order in the pair (*x*, *y*) is present, then the graph *G* will be said to be oriented (or directed), where x will be the source of the edge and y the destination. On the other side, if there is no relevant order, the graph *G* will be said to be unoriented (or undirected). In an undirected graph *G*, a clique is defined as a subgraph *G*′ = (*V*′, *E*′) of *G* with *V*′ in *V* and *E*′ in *E*, where all the pairs of nodes in *G*′ are connected by an edge. INSIdE nano is an indirect graph, where the vertices are labeled by the class of the phenotypic entities (ENM, drug, chemical and disease). The heterogeneous cliques with four (or three) different vertex classes were systematically retrieved within the network by an exhaustive search algorithm implemented in phyton (Supplementary Fig. S2).

## Validation of the Similarity Measures

The pairwise phenotypic similarities based on the tMoA were systematically compared with other independently computed similarities based on different characteristics, such as the molecular structure of the drugs and chemicals, the symptoms of the diseases, the use in clinical practice of drugs, and the pathogenic roles of chemical exposures. (See Fig. 2b). The 2D drug structures, in the form of smiles vectors, were downloaded from the DrugBank Database (https://www.drugbank.ca)^44^. Similarly, the smiles for chemical compounds were retrieved from the Chemspider Database (http://www.chemspider.com/). The pairwise drug-drug, chemical-chemical, and drug-chemical similarities were computed with the Optimal string alignment algorithm implemented in the R package “stringdist”^45^. The associations between drugs and diseases, based on clinical indications of drugs, were downloaded from the MEDI Prescription Database (https://medschool.vanderbilt.edu)^11,12^. In this case, the similarity was defined as a binary score, where 1 denotes that a given drug is used to treat a certain disease, while the 0 means no prescription indication. The associations between chemicals and diseases were downloaded from the Comparative Toxicogenomics Database (http://ctdbase.org/). The similarities between diseases were retrieved from the Supplementary materials of a previous study by Zhou *et al*.^46^, where a symptom-based human disease network was built from various databases. The comparisons between the similarity matrices derived from tMOA and the others were performed by the Mantel Test, which is used to evaluate the correlation between pairs of similarity matrices, by adopting a permutation test procedure^47^.

### Statistical evaluation of phenotypic cliques

In order to statistically validate the sets of cliques related to each disease, a permutation test was performed. The original adjacency matrix was randomly shuffled 1,000 times. For each clique, a pvalue was computed by counting how many times the strength of connection in the original clique (the sum of the weights of its edges) is higher than the strength of connection of the same clique connected by permutated edges. The obtained pvalues are then corrected with the Fdr method. Only the cliques with pvalue (<0.05) were considered.

### INSIdE nano tool

INSIdEnano is a web-based tool (publicly available at http://inano.biobyte.de) that highlights connections between phenotypic entities based on their effects on the genes. The data collection, prepossessing and integration strategies were implemented in R, as described above. The graphical tool and the routine to scan the network were implemented in Python and Javascript using the d3 library for the Graphical User Interface (GUI). INSIdE nano was developed in a client-server structure: the client is responsible for managing the user interface, collecting the user input and displaying the outputs. The server, instead, processes the data from the database according to the user inputs, and outputs the results to the client. The tool provides two different types of queries. The simple query allows the user to investigate connections of a specific element in the network. Given a node and a threshold, the tool shows all its neighbors divided into four categories: ENMs, diseases, drugs and chemicals. The conditional query allows the user to query the network by applying different filters to search for the cliques. Since the purpose of the analysis is to compare the behavior of a given element with respect to the others, the user must specify at least two different types of items. Moreover, the level of similarity necessary to report a connection between selected items, the number of items that must be in the same resulting cliques, and the number of query items being connected to the other nodes in the sub-network are requested as input. First, the tool retrieves the sub-network of all the elements, connected to the query items that satisfy the user input. Then it scans the network in search of cliques. The cliques can contain three heterogeneous elements, that will be any one of the possible combinations of three elements between ENMs, drugs, chemicals and diseases in the sub-network (e.g., an ENM, a drug, a chemical; a nano, a drug, a disease; etc.,), or they will contain exactly 4 elements (an ENM, a chemical, a drug and a disease). Those cliques are then grouped with respect to the nature of the connections between each couple of items that they contain. As a result of the analysis, the tool gives the opportunity to visualize the sub-network of all the nodes connected to the queried entities that satisfy the user requirements. It displays the list of all the cliques with the opportunity to analyze each one of them and inspect the genes underlying the connections. Moreover, direct links to relevant external sources of information are available for each phenotype. A complete tutorial is available at http://inano.biobyte.de/help.cgi and in the Supplementary materials file.

## Supplementary information

Supplementary Information

Supplementary Dataset 1

Supplementary Dataset 2

Supplementary Dataset 3

Supplementary Dataset 4

Supplementary Dataset 5

## References

[1] Krug HF (2014). Nanosafety research—are we on the right track?. Angewandte Chemie Int. Ed..

[2] Valsami-Jones E, Lynch I (2015). How safe are nanomaterials?. Science.

[3] Costa PM, Fadeel B (2016). Emerging systems biology approaches in nanotoxicology: towards a mechanism-based understanding of nanomaterial hazard and risk. Toxicol. Appl. Pharmacol..

[4] Lamb J (2006). The connectivity map: using gene-expression signatures to connect small molecules, genes, and disease. Science.

[5] Iorio F (2010). Discovery of drug mode of action and drug repositioning from transcriptional responses. Proc. Natl. Acad. Sci..

[6] Napolitano F (2013). Drug repositioning: a machine-learning approach through data integration. J. Cheminformatics.

[7] Dudley, J. T. *et al*. Computational repositioning of the anticonvulsant topiramate for inflammatory bowel Disease. Sci. *Transl*. *Medicine* **3** (2011). 10.1126/scitranslmed.3002648 PMC3479650 21849664

[8] Davis AP (2015). The comparative toxicogenomics database’s 10th year anniversary: update 2015. Nucleic Acids Res..

[9] Lamb J (2007). The connectivity map: a new tool for biomedical research. Nat. Rev. Cancer.

[10] Kong L (2013). Nanominer - integrative human transcriptomics data resource for nanoparticle research. PloS One.

[11] Wei, W.-Q., Mosley, J. D., Bastarache, L. & Denny, J. C. Validation and enhancement of a computable medication indication resource (medi) using a large practice-based dataset. In *AMIA Annual Symposium Proceedings*, vol. 2013, 1448 (American Medical Informatics Association, 2013). PMC3900157 24551419

[12] Wei W-Q (2013). Development and evaluation of an ensemble resource linking medications to their indications. J. Am. Med. Informatics Assoc..

[13] Oberdörster, G., Elder, A. & Rinderknecht, A. Nanoparticles and the brain: cause for concern? *J*. *Nanosci*. *Nanotechnol*. **9**, 4996–5007, 10.1166/jnn.2009.GR02, NIHMS150003 (2009). 10.1166/jnn.2009.gr02 PMC3804071 19928180

[14] Migliore L, Uboldi C, Di Bucchianico S, Coppedè F (2015). Nanomaterials and neurodegeneration. Environ. Mol. Mutagen..

[15] Pearson BL (2016). Identification of chemicals that mimic transcriptional changes associated with autism, brain aging and neurodegeneration. Nat. Commun..

[16] Antonini JM, Santamaria AB, Jenkins NT, Albini E, Lucchini R (2006). Fate of manganese associated with the inhalation of welding fumes: potential neurological effects. Neurotoxicology.

[17] Chin-Chan M, Navarro-Yepes J, Quintanilla-Vega B (2015). Environmental pollutants as risk factors for neurodegenerative disorders: Alzheimer and Parkinson diseases. Front. Cell. Neurosci..

[18] Shvedova AA, Kagan VE, Fadeel B (2010). Close encounters of the small kind: adverse effects of man-made materials interfacing with the nano-cosmos of biological systems. Annu. Rev. Pharmacol. Toxicol..

[19] Bastian S (2009). Toxicity of tungsten carbide and cobalt-doped tungsten carbide nanoparticles in mammalian cells *in vitro*. Environ. Heal. Perspectives.

[20] Song B, Liu J, Feng X, Wei L, Shao L (2015). A review on potential neurotoxicity of titanium dioxide nanoparticles. Nanoscale Res. Lett..

[21] Tuomela S (2013). Gene expression profiling of immune-competent human cells exposed to engineered zinc oxide or titanium dioxide nanoparticles. PloS One.

[22] Huerta-García E (2014). Titanium dioxide nanoparticles induce strong oxidative stress and mitochondrial damage in glial cells. Free. Radic. Biol. Medicine.

[23] Tilton SC (2014). Three human cell types respond to multi-walled carbon nanotubes and titanium dioxide nanobelts with cell-specific transcriptomic and proteomic expression patterns. Nanotoxicology.

[24] Buerki-Thurnherr T (2013). *In vitro* mechanistic study towards a better understanding of zno nanoparticle toxicity. Nanotoxicology.

[25] Tian L (2015). Neurotoxicity induced by zinc oxide nanoparticles: age-related differences and interaction. Sci. Reports.

[26] Xie Y, Wang Y, Zhang T, Ren G, Yang Z (2012). Effects of nanoparticle zinc oxide on spatial cognition and synaptic plasticity in mice with depressive-like behaviors. J. Biomed. Sci..

[27] Siddiqi NJ, Abdelhalim MAK, El-Ansary AK, Alhomida AS, Ong W (2012). Identification of potential biomarkers of gold nanoparticle toxicity in rat brains. J. Neuroinflammation.

[28] Schlumpf U, Meyer M, Ulrich J, Friede RL (1983). Neurologic complications induced by gold treatment. Arthritis & Rheum..

[29] Gambari P, Ostuni P, Lazzarin P, Tavolato B, Todesco S (1984). Neurotoxicity following a very high dose of oral gold (auranofin). Arthritis & Rheum..

[30] Pioro EP (2010). Dextromethorphan plus ultra low-dose quinidine reduces pseudobulbar affect. Annals neurology.

[31] Soderlund DM, Bloomquist JR (1989). Neurotoxic actions of pyrethroid insecticides. Annu. Rev. Entomol..

[32] Berggren E (2015). Chemical safety assessment using read-across: assessing the use of novel testing methods to strengthen the evidence base for decision making. Environ. Heal. Perspectives.

[33] Patlewicz G, Helman G, Pradeep P, Shah I (2017). Navigating through the minefield of read-across tools: a review of *in silico* tools for grouping. Comput. Toxicol..

[34] Gajewicz A (2017). What if the number of nanotoxicity data is too small for developing predictive nano-qsar models? An alternative read-across based approach for filling data gaps. Nanoscale.

[35] Oomen AG, Bleeker EAJ, Bos PMJ, Broekhuizen FV (2015). Grouping and read-across approaches for risk assessment of nanomaterials. Int. J. Environ. Res. Public Heal..

[36] Godwin H (2015). Nanomaterial categorization for assessing risk potential to facilitate regulatory decision-making. ACS Nano.

[37] Low Y (2013). Integrative chemical - biological read-across approach for chemical hazard classification. Chem. Res. Toxicol..

[38] Hartung T (2016). Making big sense from big data in toxicology by read-across. Altern. to Animal Exp. ALTEX.

[39] Kinaret P (2017). Network analysis reveals similar transcriptomic responses to intrinsic properties of carbon nanomaterials *in vitro* and *in vivo* network analysis reveals similar transcriptomic responses to intrinsic properties of carbon nanomaterials *in vitro* and *in vivo*. ACS Nano.

[40] Fagin, R., Kumar, R. & Sivakumar, D. Comparing top k lists. *SIAM J*. *on Discret*. *Math*. **17**, 134–160 (2003).

[41] Subramanian A (2005). Gene set enrichment analysis: a knowledge-based approach for interpreting genome-wide expression profiles. Proc. Natl. Acad. Sci. United States Am..

[42] Smirnov, N. Table for estimating the goodness of fit of empirical distributions. *The Annals Math*. *Stat*. 279–281 (1948).

[43] Napolitano F, Sirci F, Carrella D, di Bernardo D (2016). Drug-set enrichment analysis: a novel tool to investigate drug mode of action. Bioinformatics.

[44] Law V (2013). Drugbank 4.0: shedding new light on drug metabolism. Nucleic Acids Res..

[45] van der Loo M (2014). The stringdist package for approximate string matching. The R J..

[46] Zhou, X., Menche, J., Barabási, A.-L. & Sharma, A. Human symptoms–disease network. *Nat*. *Commun*. **5** (2014). 10.1038/ncomms5212 24967666

[47] Mantel N (1967). The detection of disease clustering and a generalized regression approach. Cancer Res..

