# Supplementary material for: INSIdE NANO: a systems biology framework to contextualize the mechanism-of-action of engineered nanomaterials
Source: Sci Rep. 2019 Jan 17;9:179. doi: 10.1038/s41598-018-37411-y (PMC6336851; doi:10.1038/s41598-018-37411-y)
Supplement: Supplementary file 1 — Supplementary Information [file 41598_2018_37411_MOESM1_ESM.pdf]

INSIdE NANO: a systems biology framework to contextualize the mechanism-of-action of engineered nanomaterials
Supplementary materials

Angela Serra[a,b,c], Ivica Letunic[d], Vittorio Fortino[b,c,e,f], Richard D. Handy[g], Bengt Fadeel[h], Roberto Tagliaferri[a], and Dario Greco[b,c,e,*]

[a]NeuRoNe Lab, DISA-MIS, University of Salerno, Italy
[b]Faculty of Medicine and Life Sciences, University of Tampere, Finland
[c]Institute of Biosciences and Medical Technologies, University of Tampere, Finland
[d]BioByte Solutions GmbH, Germany
[e]Institute of Biotechnology, University of Helsinki, Finland
[f]Biomedicine Institute, University of Eastern Finland, Kuopio, Finland
[g]School of Biological and Marine Sciences, University of Plymouth, United Kingdom
[h]Institute of Environmental Medicine, Karolinska Institutet, Stockholm, Sweden
[*] *To whom the correspondence should be addressed: dario.greco@staff.uta.fi*

Input Data and Preprocessing

Gene expression data of different human cell types exposed to different ENMs were downloaded from the NanoMiner Database web page [Kong et al., 2013]. NanoMiner is a collection of 634 samples derived from human primary cells and cell lines exposed to ENMs. Microarray raw data files (.CEL files, .bgx file and .txt files) coming from three technologies (Affimetrix, Illumina and Agilent) and ten chipsets (HG_Focus, HG_U133_Plus_2, HG_U133A, HG-U133A_2, HG-U219, HUGENE-1_0-st, Illumina-HumanHT-12_V3.0, Agilent-014850, Agilent-39506) were retrieved from the NanoMiner website and imported into R v 3.1.0 in order to be preprocessed by using a nested batch effect removing process.

Figure S1 shows the workflow of the pre-processing framework. First, probes of each chipset were re-annotated according to NCBI Entrez Gene Database [Maglott et al., 2005, Sayers et al., 2011]. Affimetrix probes were re-annotated by using the custom CDF packages, which were downloaded from the brainarray website [Dai et al., 2005]. Agilent probes were re-annotated by using the annotation file downloaded from the earray website (`https://earray.chem.agilent.com/earray/`) and Illumina probes were re-annotated by using the R package lumiHumanIDMapping [Du et al., 2007].

For each chipset, background correction was performed. RMA [Irizarry et al., 2003] algorithm was used for Affymetrix chipset.The bg.adjust function from affy package [Gautier et al., 2004] was used for Illumina chipset. The backgroundCorrect function, from limma package [Ritchie et al., 2015], was used for Agilent Chipset. Moreover, probe summarization was performed for each chipset by replacing replicate probes with their average value. This resulted in a separate expression data matrix for each microarray study. The re-annotated, background corrected and summarized expression values from each study were then integrated in a big data matrix of 646 samples and 7703 genes. Before removing any batch, quantile normalization was performed on the integrated data matrix to set all the distributions to the same scale. ComBat [Johnson et al., 2007] and SVA [Leek et al., 2012] were used for removing known batch effects and other unwanted variations in each microarray study. In details, ComBat was exploited to adjust each microarray study for known batch covariates, such as the dye-effect, as well as the slide and the array position. The model matrix used in ComBat included the exposure as the variable of interest and other covariates, such as the cell type, the exposure time and the dose to be preserved during the batch effect removal. Moreover, the microarray studies were also adjusted for the batch/covariate variable related to any pre-treatments. However, covariates/batches confounded with the outcome of interest were not considered. Principal component analysis (PCA) was used to investigate the

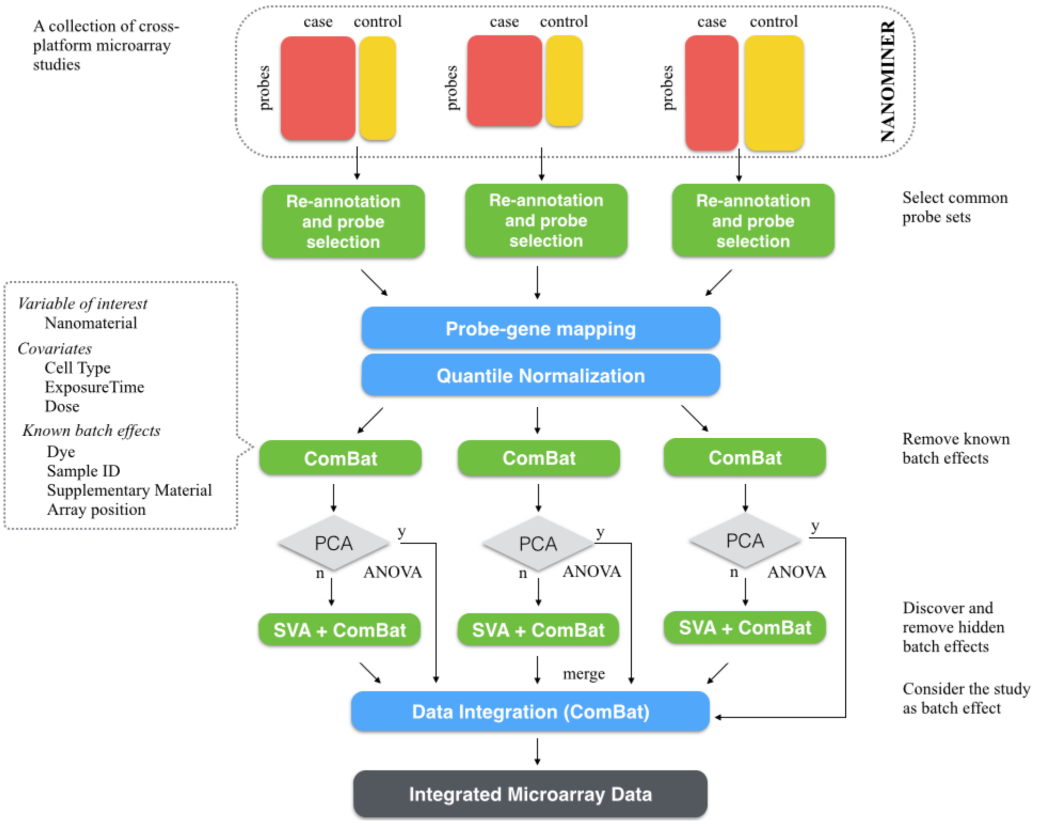

Figure S1: Outline of the microarray data integration steps at the interpretative level.

variance in each expression data matrix before and after applying ComBat, followed by ANOVA analyses to explore the associations between the covariates of interest and the most relevant components. Indeed, after removing batch effects with ComBat, if the ENM exposure variable of interest was not significantly associated to the first principal component, the SVA method was used. The SVA algorithm allows identifying and removing unknown and sources of variation while protecting the variance correlated to the variables of interest. SVA was exploited to discover batch effects, which were subsequently removed using ComBat. Gene expression data for drug treatments was downloaded from the Connectivity Map (CMap) web page [Lamb et al., 2006]. Raw data for 615 drugs was downloaded and preprocessed as in Napolitano et al [Napolitano et al., 2013]. Raw data files were quality checked in order to discard suboptimal data points. Then, the probes were re-annotated using the NCBI Entrez Gene database similarly to the process used in the NanoMiner data set, and the final matrix was normalized with the quantile method from the RMA algorithm, as described above. Next, the batch effect was estimated and removed by using the ComBat algorithm. Manually curated information about chemical-gene and disease-gene interactions was retrieved from the Comparative Toxicogenomics Database (CTD) website [Davis et al., 2015]. For each disease-gene interaction, a score representing the strength of association is provided. The distribution of these scores was investigated and used to define a threshold to filter out associations. A connection between a disease-gene and chemical-gene was considered reliable when its association strength was higher than the 95-th percentile of the overall score distribution. Disease-gene associations are not based on gene expression data. On the other hand, the connections between chemical and genes indicate whether the genes are up or down regulated by the chemicals. Moreover, only the connections between chemicals and genes from the human genome were taken into account.

```
Algorithm CliqueSearch4:
    Input: A graph G with labeled nodes (ENM, drug, chemical, disease)
    Output: list of all the heterogeneous cliques of size k=4. Each clique contains an ENM, a
    drug, a chemical and a disease.

    ENMs ← vertices of G with label ENM
    drugs ← vertices of G with label drug
    chemicals ← vertices of G with label chemical
    diseases ← vertices of G with label disease

    CliquesList ← list()

    for n in ENMs:
        drugs_n ← drugs connected to n
        for dr in drugs_n:
            chem_dr ← chemicals connected to dr
            for c in chem_dr:
                disease_c ← diseases connected to c
                for d in disease_c:
                    If (n, dr, c, d) is a clique and (n, dr, c, d) is not in CliquesList:
                    add (n, dr, c, d) to CliquesList
                    end if
                end for
            end for
        end for
    end for
    return CliquesList
```

Figure S2: The CliqueSearch4 algorithm is an exhausting search procedure that scans the INSIdE nano network to find all the cliques of four inhomogeneous elements (an ENM, a drug, a chemical and a disease).

(A)

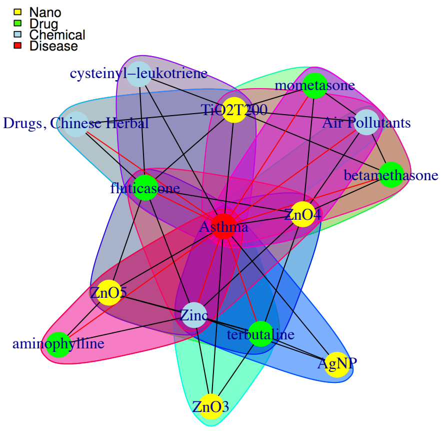

(B)

ENM	Drug(Dr)	Chemical(Chem)	Disease(Di)	ENM.Dr	ENM.Di	ENM.Chem	Dr.Di	Dr.Chem	Di.Chem	Th.
ZnO5	terbutaline	Asthma	Zinc	0.8	0.87	0.97	-0.96	-0.83	0.98	0.3
TiO2T200	fluticasone	Asthma	Drugs, Chinese Herbal	0.78	0.99	0.92	0.99	0.98	0.81	0.3
ZnO4	betamethasone	Asthma	Air Pollutants	0.91	0.75	0.93	0.81	0.7	-0.77	0.3
TiO2T200	betamethasone	Asthma	Air Pollutants	0.84	0.99	0.75	0.81	0.7	-0.77	0.3
TiO2T200	mometasone	Asthma	Air Pollutants	0.91	0.99	0.75	-0.81	-0.82	-0.77	0.3
ZnO4	fluticasone	Asthma	Zinc	1	0.75	0.99	0.99	0.88	0.98	0.3
ZnO5	fluticasone	Asthma	Zinc	0.96	0.87	0.97	0.99	0.88	0.98	0.3
TiO2T200	fluticasone	Asthma	cysteinyl-leukotriene	0.78	0.99	0.9	0.99	0.98	-0.9	0.3
ZnO3	terbutaline	Asthma	Zinc	0.92	0.85	0.81	-0.96	-0.83	0.98	0.3
ZnO4	terbutaline	Asthma	Zinc	0.76	0.75	0.99	-0.96	-0.83	0.98	0.3
AgNP	terbutaline	Asthma	Zinc	0.8	-0.79	-0.85	-0.96	-0.83	0.98	0.3
ZnO4	mometasone	Asthma	Air Pollutants	1	0.75	0.93	-0.81	-0.82	-0.77	0.3
ZnO5	aminophylline	Asthma	Zinc	0.75	0.87	0.97	0.73	0.86	0.98	0.3

Figure S3: Asthma cliques. Most relevant associations between Asthma, ENM, drugs and chemicals. Both the disease-drug and disease-chemical connections are known and represented as red edges (A). The degree of similarity (range -1, 1) between the elements of the cliques and the threshold (range 0, 1, from the most to the least stringent) used for the analysis are reported (B).

(A)

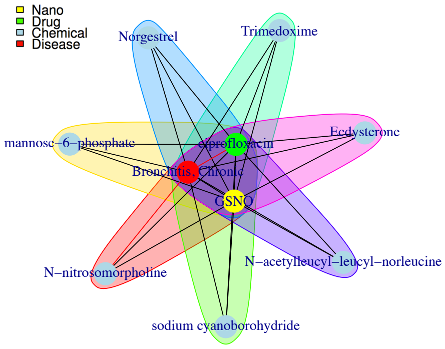

(B)

ENM	Drug(Dr)	Chemical(Chem)	Disease(Di)	ENM.Dr	ENM.Di	ENM.Chem	Dr.Di	Dr.Chem	Di.Chem	Th.
GSNO	ciprofloxacin	Bronchitis, Chronic	N-nitrosomorpholine	0.87	-0.93	0.9	-0.9	0.9	-0.93	0.2
GSNO	ciprofloxacin	Bronchitis, Chronic	mannose-6-phosphate	0.87	-0.93	0.91	-0.9	0.95	-0.92	0.2
GSNO	ciprofloxacin	Bronchitis, Chronic	sodium cyanoborohydride	0.87	-0.93	0.93	-0.9	0.95	-0.95	0.2
GSNO	ciprofloxacin	Bronchitis, Chronic	Trimedoxime	0.87	-0.93	0.91	-0.9	0.91	0.92	0.2
GSNO	ciprofloxacin	Bronchitis, Chronic	Norgestrel	0.87	-0.93	0.99	-0.9	0.88	-0.92	0.2
GSNO	ciprofloxacin	Bronchitis, Chronic	N-acetylleucyl-leucyl-norleucine	0.87	-0.93	0.92	-0.9	0.92	0.95	0.2
GSNO	ciprofloxacin	Bronchitis, Chronic	Ecdysterone	0.87	-0.93	-0.88	-0.9	0.89	-0.89	0.2

Figure S4: Bronchitis Chronic cliques. Most relevant associations between Bronchitis Chronic, ENM, drugs and chemicals. Both the disease-drug and disease-chemical connections are known and represented as red edges (A). The degree of similarity (range -1, 1) between the elements of the cliques and the threshold (range 0, 1, from the most to the least stringent) used for the analysis are reported (B).

(A)

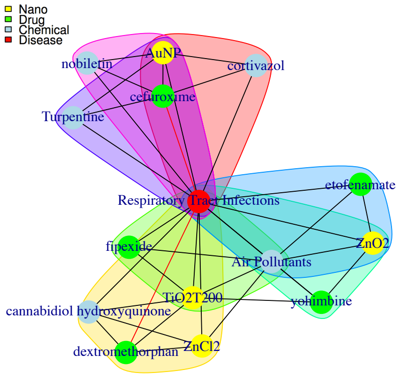

(B)

ENM	Drug(Dr)	Chemical(Chem)	Disease(Di)	ENM.Dr	ENM.Di	ENM.Chem	Dr.Di	Dr.Chem	Di.Chem	Th.
AuNP	cefuroxime	Respiratory Tract Infections	cortivazol	0.91	0.92	0.95	-0.99	-0.99	-0.87	0.3
ZnCl2	dextromethorphan	Respiratory Tract Infections	cannabidiol hydroxyquinone	0.61	0.82	0.79	-0.99	-0.89	0.85	0.3
TiO2T200	fipexide	Respiratory Tract Infections	Air Pollutants	0.85	0.95	0.75	-0.99	-0.89	-0.79	0.3
ZnO2	yohimbine	Respiratory Tract Infections	Air Pollutants	0.88	-0.7	-0.9	0.75	0.91	-0.79	0.3
ZnO2	etofenamate	Respiratory Tract Infections	Air Pollutants	0.94	-0.7	-0.9	0.82	0.79	-0.79	0.3
AuNP	cefuroxime	Respiratory Tract Infections	Turpentine	0.91	0.92	0.96	-0.99	-0.99	0.77	0.3
AuNP	cefuroxime	Respiratory Tract Infections	nobiletin	0.91	0.92	0.9	-0.99	-0.9	-0.84	0.3

Figure S5: Respiratory Tract Infections cliques. Most relevant associations between Respiratory Tract Infections, ENM, drugs and chemicals. Both the disease-drug and disease-chemical connections are known and represented as red edges (A). The degree of similarity (range -1, 1) between the elements of the cliques and the threshold (range 0, 1, from the most to the least stringent) used for the analysis are reported (B).

(A)

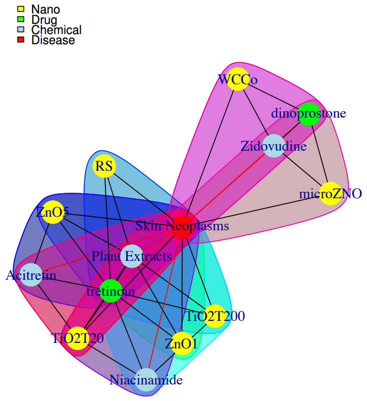

(B)

ENM	Drug(Dr)	Chemical(Chem)	Disease(Di)	ENM.Dr	ENM.Di	ENM.Chem	Dr.Di	Dr.Chem	Di.Chem	Th.
ZnO5	tretinoin	Skin Neoplasms	Plant Extracts	0.82	0.96	0.83	0.93	-0.95	-0.92	0.3
ZnO5	tretinoin	Skin Neoplasms	Acitretin	0.82	0.96	-0.77	0.93	0.64	0.83	0.3
TiO2T20	tretinoin	Skin Neoplasms	Acitretin	0.83	0.95	0.99	0.93	0.64	0.83	0.3
TiO2T20	tretinoin	Skin Neoplasms	Niacinamide	0.83	0.95	0.86	0.93	0.85	0.82	0.3
ZnO1	tretinoin	Skin Neoplasms	Plant Extracts	0.73	0.7	0.62	0.93	-0.95	-0.92	0.4
microZNO	dinoprostone	Skin Neoplasms	Zidovudine	0.59	0.75	-0.68	0.92	0.88	0.9	0.4
TiO2T200	tretinoin	Skin Neoplasms	Plant Extracts	0.77	0.65	0.99	0.93	-0.95	-0.92	0.4
RS	tretinoin	Skin Neoplasms	Plant Extracts	0.88	0.75	0.6	0.93	-0.95	-0.92	0.4
ZnO5	tretinoin	Skin Neoplasms	Plant Extracts	0.82	0.96	0.83	0.93	-0.95	-0.92	0.4
TiO2T200	tretinoin	Skin Neoplasms	Niacinamide	0.77	0.65	0.78	0.93	0.85	0.82	0.4
TiO2T20	tretinoin	Skin Neoplasms	Niacinamide	0.83	0.95	0.86	0.93	0.85	0.82	0.4
ZnO5	tretinoin	Skin Neoplasms	Acitretin	0.82	0.96	-0.77	0.93	0.64	0.83	0.4
WCCo	dinoprostone	Skin Neoplasms	Zidovudine	0.63	0.64	0.92	0.92	0.88	0.9	0.4
TiO2T20	tretinoin	Skin Neoplasms	Acitretin	0.83	0.95	0.99	0.93	0.64	0.83	0.4
TiO2T20	tretinoin	Skin Neoplasms	Plant Extracts	0.83	0.95	0.77	0.93	-0.95	-0.92	0.4

Figure S6: Skin Neoplasms cliques. Most relevant associations between Skin Neoplasms, ENM, drugs and chemicals. Both the disease-drug and disease-chemical connections are known and represented as red edges (A). The degree of similarity (range -1, 1) between the elements of the cliques and the threshold (range 0, 1, from the most to the least stringent) used for the analysis are reported (B).

(A)

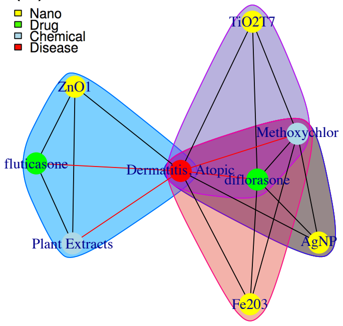

(B)

ENM	Drug(Dr)	Chemical(Chem)	Disease(Di)	ENM.Dr	ENM.Di	ENM.Chem	Dr.Di	Dr.Chem	Di.Chem	Th.
AgNP	diflorasone	Dermatitis, Atopic	Methoxychlor	-0.99	0.81	0.94	0.94	0.92	0.88	0.3
Fe203	diflorasone	Dermatitis, Atopic	Methoxychlor	-1	0.94	-0.74	0.94	0.92	0.88	0.4
AgNP	diflorasone	Dermatitis, Atopic	Methoxychlor	-0.99	0.81	0.94	0.94	0.92	0.88	0.4
TiO2T7	diflorasone	Dermatitis, Atopic	Methoxychlor	-0.99	0.93	0.81	0.94	0.92	0.88	0.4
ZnO1	fluticasone	Dermatitis, Atopic	Plant Extracts	0.77	-0.71	0.62	0.76	0.99	-0.93	0.4

Figure S7: Dermatitis Atopic cliques. Most relevant associations between Dermatitis Atopic, ENM, drugs and chemicals. Both the disease-drug and disease-chemical connections are known and represented as red edges (A). The degree of similarity (range -1, 1) between the elements of the cliques and the threshold (range 0, 1, from the most to the least stringent) used for the analysis are reported (B).

(A)

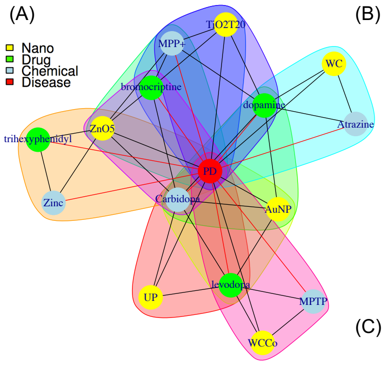

(B)

ENM	Drug(Dr)	Chemical(Chem)	Disease(Di)	ENM.Dr	ENM.Di	ENM.Chem	Dr.Di	Dr.Chem	Di.Chem	Th.
UP	levodopa	Carbidopa	PD	0.85	0.91	-0.92	0.9	0.97	-0.71	0.3
ZnO5	trihexyphenidyl	Zinc	PD	0.96	0.74	0.97	0.83	0.94	0.98	0.3
AuNP	levodopa	Carbidopa	PD	0.92	0.9	0.94	0.9	0.97	-0.71	0.3
AuNP	dopamine	Carbidopa	PD	1	0.9	0.94	0.89	0.72	-0.71	0.3
ZnO5	bromocriptine	MPP+	PD	0.95	0.74	0.95	-0.98	0.93	-0.97	0.3
WC	dopamine	Atrazine	PD	0.8	0.91	0.81	0.89	-0.99	0.97	0.3
TiO2T20	bromocriptine	MPP+	PD	0.9	0.93	0.89	-0.98	0.93	-0.97	0.3
TiO2T20	dopamine	MPP+	PD	0.89	0.93	0.89	0.89	-0.84	-0.97	0.3
ZnO5	bromocriptine	Carbidopa	PD	0.95	0.74	0.96	-0.98	0.89	-0.71	0.3
WCCo	levodopa	MPTP	PD	0.75	0.65	0.99	0.9	0.77	0.89	0.4

(C)

ENM	Drug(Dr)	Chamical(Chem)	Disease(Di)	Genes
UP	levodopa	Carbidopa	PD	GSK3B (-/+); RPS6KB1 (-/+);
ZnO5	trihexyphenidyl	Zinc	PD	ATP5A1 (-/+); DRD4 (+/-); MAPT (+/+); ATF3 (+/-); IRS2 (-/-); NPY (+/-);
AuNP	levodopa	Carbidopa	PD	RELA (+/-); MCL1 (-/-); RPS6KB1 (-/+);
AuNP	dopamine	Carbidopa	PD	AKT1 (+/-); RELA (+/-); GRIN1 (+/+); FOS (-/+); MCL1 (+/-); RPS6KB1 (+/+);
ZnO5	bromocriptine	MPP+	PD	APOE (-/-); KCNJ6 (-/+); MAPT (-/-); EGR1 (+/+); FOS (-/-); ATF3 (+/+);
WC	dopamine	Atrazine	PD	AKT1 (+/-); ZFP36 (+/+); MAPK9 (+/-); HTR1A (-/-); RPS6KB1 (+/-); CASP3 (+/+);
TiO2T20	bromocriptine	MPP+	PD	BDNF (-/+); GCLM (-/-); ODC1 (+/+); EGR1 (+/+); CP (+/-); BCL2 (+/+);
TiO2T20	dopamine	MPP+	PD	CTNNB1 (+/+); GCLM (+/+); ODC1 (-/+); NUPR1 (-/+); GRIN1 (-/+); MYC (+/-);
ZnO5	bromocriptine	Carbidopa	PD	GRIN1 (-/+); FOS (+/+);
WCCo	levodopa	MPTP	PD	CASP7 (-/-); NGF (-/-); NEFH (+/-); TACC3 (+/+); CASP3 (+/-); RAF1 (+/-);

Figure S8: Parkinson Disease cliques. Most relevant associations between Parkinson Disease (PD), ENM, drugs and chemicals. Both the disease-drug and disease-chemical connections are known and represented as red edges (A). For each clique, a list of relevant genes characterizing the connections between its elements are reported (B). The font color of the gene symbols (red for up-regulation and green for down-regulation) indicates the direction of expression perturbation exacerbated by ENM exposure. The symbols in brackets ("+" for up-regulation and "-" for down-regulation) separated by "/" indicate the effect on the gene expression by the drug and the chemical, respectively. The degree of similarity (range -1, 1) between the elements of the cliques and the threshold (range 0, 1, from the most to the least stringent) used for the analysis are reported (C).

(A)

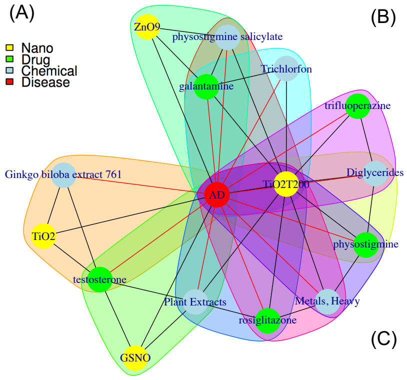

(B)

ENM	Drug(Dr)	Chemical(Chem)	Disease(Di)	ENM.Dr	ENM.Di	ENM.Chem	Dr.Di	Dr.Chem	Di.Chem	Th.
TiO2T200	galantamine	physostigmine salicylate	AD	0.95	0.74	0.78	0.82	0.9	0.92	0.3
TiO2	testosterone	Ginkgo biloba extract 761	AD	0.78	0.9	0.94	0.95	-0.89	-0.97	0.3
TiO2T200	physostigmine	Diglycerides	AD	0.87	0.74	0.8	0.86	-0.98	0.78	0.3
GSNO	testosterone	Plant Extracts	AD	0.84	0.99	0.97	0.95	0.98	-0.93	0.3
ZnO9	galantamine	physostigmine salicylate	AD	0.94	0.72	0.79	0.82	0.9	0.92	0.3
TiO2T200	galantamine	Trichlorfon	AD	0.95	0.74	0.91	0.82	0.96	0.87	0.3
TiO2T200	rosiglitazone	Plant Extracts	AD	0.89	0.74	0.99	-0.92	0.92	-0.93	0.3
TiO2T200	physostigmine	Metals, Heavy	AD	0.87	0.74	0.88	0.86	-0.88	-0.9	0.3
TiO2T200	trifluoperazine	Diglycerides	AD	0.96	0.74	0.8	-0.83	0.91	0.78	0.3
TiO2T200	rosiglitazone	Metals, Heavy	AD	0.89	0.74	0.88	-0.92	0.9	-0.9	0.3

(C)

ENM	Drug(Dr)	Chamical(Chem)	Disease(Di)	Genes
TiO2T200	galantamine	physostigmine salicylate	AD	CYP1A1 (+/+); IGF1R (+/-); VCAM1 (+/-); TNF (+/-); ALB (-/-); CSF2 (-/-);
TiO2	testosterone	Ginkgo biloba extract 761	AD	HIF1A (+/-); GCLM (+/-); THBS1 (-/-); HMOX1 (+/-); FOS (+/-); AKT1 (+/-);
TiO2T200	physostigmine	Diglycerides	AD	CYP1A2 (+/+); GSTA4 (+/+); NOS1 (+/+); CYP1A1 (-/-);
GSNO	testosterone	Plant Extracts	AD	ATF3 (+/-); CEBPB (+/-); TNF (+/-); PLAUR (+/-); HMGCR (+/-); SPP1 (+/-);
ZnO9	galantamine	physostigmine salicylate	AD	EPO (+/-); ATP5A1 (+/+); PDGFRB (+/+); HMOX1 (-/-); TNF (-/-); DOF3 (-/-);
TiO2T200	galantamine	Trichlorfon	AD	FABP4 (+/+); CYP1A1 (+/+); MAPK1 (+/-); IGF1 (-/+); TNF (-/+); CD36 (-/-);
TiO2T200	rosiglitazone	Plant Extracts	AD	TXNIP (-/-); STAT3 (+/-); MKI67 (+/-); SPP1 (+/-); MYC (+/-); VEGFA (-/-);
TiO2T200	physostigmine	Metals, Heavy	AD	BDNF (+/-); CASP9 (+/-); CYP1A1 (+/+); BCL2 (-/-); TNF (+/-); ALB (-/+);
TiO2T200	trifluoperazine	Diglycerides	AD	CYP1A2 (-/+); GSTA4 (+/+); NOS1 (-/+);
TiO2T200	rosiglitazone	Metals, Heavy	AD	BDNF (+/+); CYP1A1 (+/-); FOS (+/-); MYC (-/-); CXCL8 (+/+); TNFAIP3 (+/+);

Figure S9: Alzheimer Disease cliques. Most relevant associations between Alzheimer Disease (AD), ENM, drugs and chemicals. Both the disease-drug and disease-chemical connections are known and represented as red edges (A). For each clique, a list of relevant genes characterizing the connections between its elements are reported (B). The font color of the gene symbols (red for up-regulation and green for down-regulation) indicates the direction of expression perturbation exacerbated by ENM exposure. The symbols in brackets ("+" for up-regulation and "-" for down-regulation) separated by "/" indicate the effect on the gene expression by the drug and the chemical, respectively. The degree of similarity (range -1, 1) between the elements of the cliques and the threshold (range 0, 1, from the most to the least stringent) used for the analysis are reported (C).

(A)

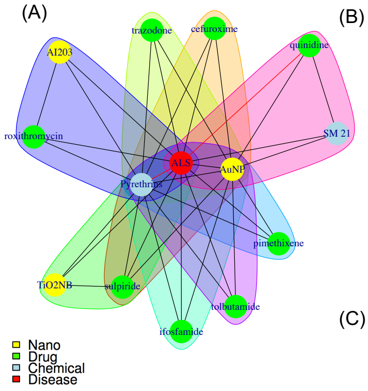

(B)

ENM	Drug(Dr)	Chemical(Chem)	Disease(Di)	ENM.Dr	ENM.Di	ENM.Chem	Dr.Di	Dr.Chem	Di.Chem	Th.
AuNP	sulpiride	Pyrethrins	ALS	0.97	0.96	0.95	0.98	0.95	0.88	0.2
AuNP	cefuroxime	Pyrethrins	ALS	0.91	0.96	0.95	-0.91	-0.99	0.88	0.3
AuNP	trazodone	Pyrethrins	ALS	0.96	0.96	0.95	-0.89	-0.96	0.88	0.3
TiO2NB	sulpiride	Pyrethrins	ALS	0.99	-0.95	0.81	0.98	0.95	0.88	0.3
AuNP	ifosfamide	Pyrethrins	ALS	0.89	0.96	0.95	0.85	0.75	0.88	0.3
AuNP	pimethixene	Pyrethrins	ALS	0.96	0.96	0.95	-0.77	-0.86	0.88	0.3
AI203	roxithromycin	Pyrethrins	ALS	0.73	0.81	0.9	-0.98	0.95	0.88	0.3
AuNP	tolbutamide	Pyrethrins	ALS	0.97	0.96	0.95	-0.81	-0.8	0.88	0.3
AuNP	quinidine	SM 21	ALS	0.97	0.96	0.84	-0.72	-0.86	-0.94	0.3

(C)

ENM	Drug(Dr)	Chemical(Chem)	Disease(Di)	Genes
AuNP	sulpiride	Pyrethrins	ALS	HMOX1 (-/+); ALDOA (+/+); PSMB3 (+/+); PSMC4 (+/+); PRDX2 (+/-); TRAF2 (-/-);
AuNP	cefuroxime	Pyrethrins	ALS	ERO1A (+/+); ALDOA (+/+); PSMB3 (+/+); PRDX2 (-/-);
AuNP	trazodone	Pyrethrins	ALS	HMOX1 (+/+); PSMB3 (-/+); PRDX2 (-/-); TRAF2 (+/-);
TiO2NB	sulpiride	Pyrethrins	ALS	HMOX1 (-/+); UBE4B (+/+); PSMC4 (+/+); TRAF2 (-/-); GSR (+/+);
AuNP	ifosfamide	Pyrethrins	ALS	HMOX1 (-/+); ERO1A (-/+); PSMB3 (+/+); PSMC4 (+/+); PRDX2 (+/-); TRAF2 (+/-);
AuNP	pimethixene	Pyrethrins	ALS	HMOX1 (+/+); ERO1A (-/+); PRDX2 (-/-);
AI203	roxithromycin	Pyrethrins	ALS	HMOX1 (-/+); BAX (-/+); UBE4B (-/+);
AuNP	tolbutamide	Pyrethrins	ALS	HMOX1 (+/+); ERO1A (-/+); ALDOA (+/+); PSMB3 (+/+); PSMC4 (+/+); TRAF2 (+/-);
AuNP	quinidine	SM 21	ALS	HMOX1 (+/-); ERO1A (-/-); ITGAM (-/+);

Figure S10: Amyotrophic Lateral Sclerosis cliques. Most relevant associations between Amyotrophic Lateral Sclerosis (ALS), ENM, drugs and chemicals. Both the disease-drug and disease-chemical connections are known and represented as red edges (A). For each clique, a list of relevant genes characterizing the connections between its elements are reported (B). The font color of the gene symbols (red for up-regulation and green for down-regulation) indicates the direction of expression perturbation exacerbated by ENM exposure. The symbols in brackets ("+" for up-regulation and "-" for down-regulation) separated by "/" indicate the effect on the gene expression by the drug and the chemical, respectively. The degree of similarity (range -1, 1) between the elements of the cliques and the threshold (range 0, 1, from the most to the least stringent) used for the analysis are reported (C).

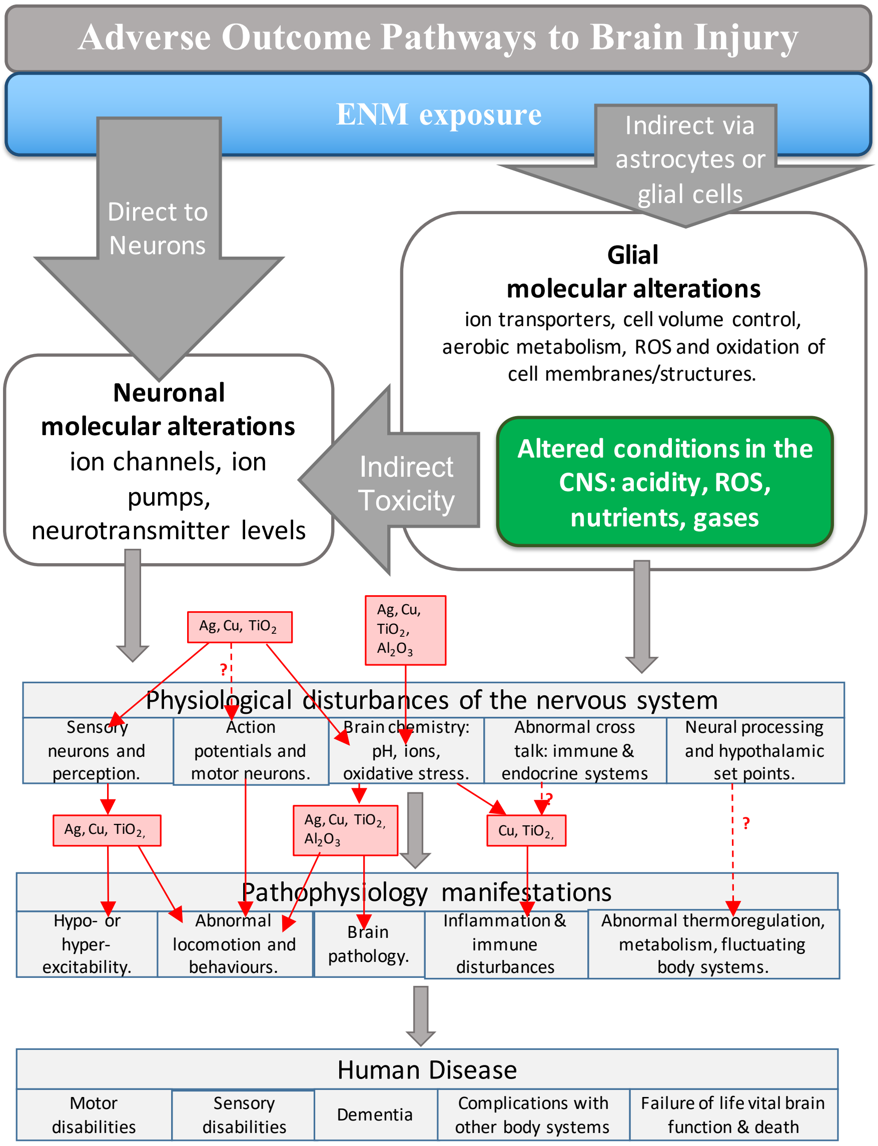

Figure S11: Adverse outcome pathway (AOP) for brain injury and the role of ENMs. The metal-containing ENMs involvement (red boxes and arrows) causes biochemical disturbances either targeting directly the neural cells, or indirectly interfering with the functions of the glial cells.

References

[Dai et al., 2005] Dai, M., Wang, P., Boyd, A. D., Kostov, G., Athey, B., Jones, E. G., Bunney, W. E., Myers, R. M., Speed, T. P., Akil, H., et al. (2005). Evolving gene/transcript definitions significantly alter the interpretation of genechip data. *Nucleic Acids Research*, 33(20):e175–e175.

[Davis et al., 2015] Davis, A. P., Grondin, C. J., Lennon-Hopkins, K., Saraceni-Richards, C., Sciaky, D., King, B. L., Wiegers, T. C., and Mattingly, C. J. (2015). The comparative toxicogenomics database's 10th year anniversary: update 2015. *Nucleic Acids Research*, 43(D1):D914–D920.

[Du et al., 2007] Du, P., Kibbe, W. A., and Lin, S. M. (2007). nuid: a universal naming scheme of oligonucleotides for illumina, affymetrix, and other microarrays. *Biology Direct*, 2(1):16.

[Gautier et al., 2004] Gautier, L., Cope, L., Bolstad, B. M., and Irizarry, R. A. (2004). Affy - analysis of affymetrix genechip data at the probe level. *Bioinformatics*, 20(3):307–315.

[Irizarry et al., 2003] Irizarry, R. A., Hobbs, B., Collin, F., Beazer-Barclay, Y. D., Antonellis, K. J., Scherf, U., Speed, T. P., et al. (2003). Exploration, normalization, and summaries of high density oligonucleotide array probe level data. *Biostatistics*, 4(2):249–264.

[Johnson et al., 2007] Johnson, W. E., Li, C., and Rabinovic, A. (2007). Adjusting batch effects in microarray expression data using empirical bayes methods. *Biostatistics*, 8(1):118–127.

[Kong et al., 2013] Kong, L., Tuomela, S., Hahne, L., Ahlfors, H., Yli-Harja, O., Fadeel, B., Lahesmaa, R., and Autio, R. (2013). Nanominer - integrative human transcriptomics data resource for nanoparticle research. *PloS ONE*, 8(7):e68414.

[Lamb et al., 2006] Lamb, J., Crawford, E. D., Peck, D., Modell, J. W., Blat, I. C., Wrobel, M. J., Lerner, J., Brunet, J.-P., Subramanian, A., Ross, K. N., et al. (2006). The connectivity map: using gene-expression signatures to connect small molecules, genes, and disease. *Science*, 313(5795):1929–1935.

[Leek et al., 2012] Leek, J. T., Johnson, W. E., Parker, H. S., Jaffe, A. E., and Storey, J. D. (2012). The sva package for removing batch effects and other unwanted variation in high-throughput experiments. *Bioinformatics*, 28(6):882–883.

[Maglott et al., 2005] Maglott, D., Ostell, J., Pruitt, K. D., and Tatusova, T. (2005). Entrez gene: gene-centered information at ncbi. *Nucleic Acids Research*, 33(suppl 1):D54–D58.

[Napolitano et al., 2013] Napolitano, F., Zhao, Y., Moreira, V. M., Tagliaferri, R., Kere, J., D'Amato, M., and Greco, D. (2013). Drug repositioning: a machine-learning approach through data integration. *J. Cheminformatics*, 5:30.

[Ritchie et al., 2015] Ritchie, M. E., Phipson, B., Wu, D., Hu, Y., Law, C. W., Shi, W., and Smyth, G. K. (2015). Limma powers differential expression analyses for RNA-sequencing and microarray studies. *Nucleic Acids Research*, 43(7):e47.

[Sayers et al., 2011] Sayers, E. W., Barrett, T., Benson, D. A., Bolton, E., Bryant, S. H., Canese, K., Chetvernin, V., Church, D. M., DiCuccio, M., Federhen, S., et al. (2011). Database resources of the national center for biotechnology information. *Nucleic Acids Research*, 39(suppl 1):D38–D51.

INSIdE NANO: a systems biology framework to contextualize the mechanism-of-action of engineered nanomaterials

Angela Serra[a,b,c], Ivica Letunic[d], Vittorio Fortino[b,c,e,f], Richard D. Handy[g], Bengt Fadeel[h], Roberto Tagliaferri[a], and Dario Greco[b,c,e,*]

[a]NeuRoNe Lab, DISA-MIS, University of Salerno, Italy
[b]Faculty of Medicine and Life Sciences, University of Tampere, Finland
[c]Institute of Biosciences and Medical Technologies, University of Tampere, Finland
[d]BioByte Solutions GmbH, Germany
[e]Institute of Biotechnology, University of Helsinki, Finland
[f]Biomedicine Institute, University of Eastern Finland, Kuopio, Finland
[g]School of Biological and Marine Sciences, University of Plymouth, United Kingdom
[h]Institute of Environmental Medicine, Karolinska Institutet, Stockholm, Sweden

Supplementary Table S1

Table S1. Example data of statistically significant effects of engineered nanomaterials (ENMs) on brain and behaviours of fish during *in vivo* exposures and the relevant metal salt or bulk material.

ENM (primary size)	Species, exposure route and duration	Accumulation in whole brain	Disturbances to biochemistry in whole brain homogenates	Pathologies	Behavioural effects	Evidence of hypoxia?	Reference
Cu NPs (87 nm)	Rainbow trout (Respiratory exposure, 0.5 days)	Exposure of peripheral sensory systems intended without Cu accumulation in the brain itself.	↓ ratio GSH:GSSG (0.72)[#1]<br>No effect on GSH:GSSG (0.98)	No pathology of the olfactory rosette.	↓ alarm response	No, short exposure not intended as an internal dose.	Sovová, T et al. (2014)
$CuSO_4$				clear degradation of sensory cilia surrounding the midline raphe.	↓ exploratory behaviours		
Cu NPs (20 nm)	Rainbow trout. Respiratory exposure to 50 µg l^{-1} for 12	No detectable total [Cu] increases against existing background.	↓ TBARS (0.56)[2]<br>↓ Na^+,K^+-ATPase activity (0.78)	↑ incidences of enlarged nerve cells in telencephalon (3.4	No signs of aggression or unusual swimming behaviours in	No, but some ↑ gill pathologies including mild oedema, swollen	Shaw, B. J et

	hours.			Cu NPs; 3.2 $CuSO_4$); necrotic cell bodies (26.3 Cu NPs; 17.5 $CuSO_4$); and enlarged blood vessels on ventral surface of cerebellum.	surviving fish.	mucocytes. Some loss of plasma Na^+.	al. (2012)
$CuSO_4$			↓ TBARS (0.38)<br>↓ Na^+,K^+-ATPase activity (0.66)			No, but some ↑ gill pathologies and some loss of plasma Na^+.	
TiO_2-NPs (P25 particles, 21 nm)	Rainbow trout (respiratory, 14 days)	Trend of increasing total [Ti], but not statistically significant against existing background.	↑ GSH (1.2)$^{\#}$<br>↑ TBARS (1.7)$^{\#}$<br>↑ [K] (1.5)$^{\#}$<br>↑ [Zn] (1.6)$^{\#}$<br>↑ [Ca] (1.58)	Abnormal blood vessels on surface of cerebellum.	↓ spontaneous movements at high velocities (0.22)	↑ Gill pathologies, ionoregulatory perturbations and ↑ RBC recruitment into blood[3]	Boyle, D. et al. (2013)
TiO_2-bulk powder			↑ GSH (1.1)<br>[K] (0.93)<br>[Zn] (0.98)<br>↑ [Ca] (1.94)	Abnormal blood vessels on surface of cerebellum	movements at high velocities (0.32)	Gill pathologies, and ↑ RBC recruitment[3]	
TiO_2-NPs P25 particles (24 nm)	Rainbow trout. Respiratory exposure to 0.1, 0.5 and 1 mg l^{-1} for 14 days.	↑ [Ti] (2.0) by day 7 in the 0.5 mg l^{-1} exposure group.	↑ TBARS (2.22)$^{\#}$<br>Transient perturbations in brain [K], [Zn] and [Cu]	No evidence of gross inflammation of the dura or cranial bleeding. Fish in the 0.1 mg l^{-1} TiO_2 ENM treatment showing necrotic cell bodies in the cerebrum.	Atypical loss of buoyancy control observed.	Gill pathologies accompanied by transient ionoregulatory disturbances and oxidative stress (↑ TBARS and ↑ GSH).	Federici, G. et al. (2007)
TiO_2 (80 and 25 nm)	CD-1 mice. Single 'injection' in the gut with 5 g/kg body weight using 0.5% hydroxypropy lmethylcellulo se as vehicle.	↑Total [Ti] in whole brain with the 80 nm treatment (~5 fold).	Only blood chemistry measured but red cells contained TiO_2 and this contribute to the apparent load in whole brain analysis.	Vacuoles in the hippocampus in animals from the 80 nm particle treatment.	Not reported	No, but some elevations in total Ti in the lung after the injection.	Wang, J. et al. (2007)

Aluminium oxide NPs (< 100 nm)	Sprague Dawley rats injected with 1 mg/kg or 50 mg/kg every other day for up to 60 days.	No reported	Not reported, the focus of the paper is immunohistochemistry.	Activation of glial cells inferred from immunohistochemistry. Increased staining for Glial fibrillary acidic protein (GFAP) and nestin in astrocytes of brain cortex and hippocampus. Astrocytes are enlarged with thin, shortened processes.	Not reported	Not reported	Li, X. bo et al. (2009)

[#]Indicates nano-scale specific effect

[1]Values in parentheses show the magnitude of a significant effect relative to the control

[2]At 20 µg L^{-1}, equal to Cu (as $CuSO_4$)

[3]Intravenous injection of TiO_2 caused no effect on brains of fish (Boyle et al. 2013b)

[4]AChE activity in brains unaffected by NPs exposure

References

Boyle, D., Al-Bairuty, G. A., Ramsden, C. S., Sloman, K. A., Henry, T. B., & Handy, R. D. (2013). Subtle alterations in swimming speed distributions of rainbow trout exposed to titanium dioxide nanoparticles are associated with gill rather than brain injury. *Aquatic Toxicology*, *126*, 116–127. https://doi.org/10.1016/j.aquatox.2012.10.006

Federici, G., Shaw, B. J., & Handy, R. D. (2007). Toxicity of titanium dioxide nanoparticles to rainbow trout (Oncorhynchus mykiss): Gill injury, oxidative stress, and other physiological effects. *Aquatic Toxicology*, *84*(4), 415–430. https://doi.org/10.1016/j.aquatox.2007.07.009

Li, X. bo, Zheng, H., Zhang, Z. ren, Li, M., Huang, Z. yong, Schluesener, H. J., … Xu, S. qing. (2009). Glia activation induced by peripheral administration of aluminum oxide nanoparticles in rat brains. *Nanomedicine: Nanotechnology, Biology, and Medicine*, *5*(4), 473–479. https://doi.org/10.1016/j.nano.2009.01.013

Shaw, B. J., Al-Bairuty, G., & Handy, R. D. (2012). Effects of waterborne copper nanoparticles and copper sulphate on rainbow trout, (Oncorhynchus mykiss): Physiology and accumulation. *Aquatic Toxicology*, *116–117*(March), 90–101. https://doi.org/10.1016/j.aquatox.2012.02.032

Sovová, T., Boyle, D., Sloman, K. A., Vanegas Pérez, C., & Handy, R. D. (2014). Impaired behavioural response to alarm substance in rainbow trout exposed to copper nanoparticles. *Aquatic Toxicology*, *152*, 195–204. https://doi.org/10.1016/j.aquatox.2014.04.003

Wang, J., Zhou, G., Chen, C., Yu, H., Wang, T., Ma, Y., … Chai, Z. (2007). Acute toxicity and biodistribution of different sized titanium dioxide particles in mice after oral administration. *Toxicology Letters*, *168*(2), 176–185. https://doi.org/10.1016/j.toxlet.2006.12.001

INSIdE NANO: a systems biology framework to contextualize the mechanism-of-action of engineered nanomaterials

Angela Serra[a,b,c], Ivica Letunic[d], Vittorio Fortino[b,c,e,f], Richard D. Handy[g], Bengt Fadeel[h], Roberto Tagliaferri[a], and Dario Greco[b,c,e,*]

[a]NeuRoNe Lab, DISA-MIS, University of Salerno, Italy
[b]Faculty of Medicine and Life Sciences, University of Tampere, Finland
[c]Institute of Biosciences and Medical Technologies, University of Tampere, Finland
[d]BioByte Solutions GmbH, Germany
[e]Institute of Biotechnology, University of Helsinki, Finland
[f]Biomedicine Institute, University of Eastern Finland, Kuopio, Finland
[g]School of Biological and Marine Sciences, University of Plymouth, United Kingdom
[h]Institute of Environmental Medicine, Karolinska Institutet, Stockholm, Sweden

Supplementary information in support of the figure on the adverse outcome pathways to brain injury.

The tables below illustrate how the genes reported in this study (the genes listed in Table S6) can be placed in the AOP for brain injury; especially the direct molecular events in the nerves and the indirect molecular events in astrocytes/glial cells that alter the housekeeping in the environment of the central nervous system. Genes that are known to express when pathology is already present are also shown. Three example 'case studies' are made on different nanomaterials to illustrate how the genes could be included in the adverse outcome pathways in the different diseases. The 'possible adverse outcome' in each table indicates from a physiological/pathological perspective the main functional consequences of the gene expression that has changed (not intended as an exhaustive list).

Note for each table below: (-), downregulated; (+), upregulated;

Table S2 Gene expression changes associated with the steps in the adverse outcome pathway (AOP) for TiO2T20 in Parkinson's disease.

**Genes and their products**	**Possible adverse outcome**
Direct to the nerve cell.	
KCNJ6 (-); voltage-gated K^+ channel, downregulated	Decrease of K^+ flux in the nerve and loss of excitability.
TAC1 (-); encodes neurokinin neurotransmitter	Impaired neural pathways, especially effectors to smooth muscle.

S100A4 (+); calcium binding protein, upregulated	Altered availability of calcium to synapses and synaptic transmission.
GRIN1 (+); encodes part of the NMDA receptors in the hypothalamus and elsewhere.	Synthesis of new receptors as NMDA receptor functions are already impaired; such as long term potentiation and memory formation.
NEFH (-); encodes neurofilament heavy polypeptide.	Axons and other nerve filaments prone to injury.
Indirect effects on glial cells and housekeeping of the CNS environment.	
TMP3 (-); metalloprotease, downregulated.	Renewal of extracellular matrix in the brain tissue impaired.
ODC1 (-); ornithine decarboxylase, downregulated.	Slowed ornithine cycle with potential to reduce protein turnover. The latter essential for cell repair.
GCLM and GCLC (-) sub-units of glutamate cysteine ligase needed for GSH synthesis are downregulated.	Loss of GSH chemical antioxidant defence from the tissue. Loss of GSH as a copper chelator.
CCL2 (+); chemokine attractant precursor.	Initiation of early steps in inflammation.
VEGFA (+); gene for endothelial growth factor upregulated.	Repair of vasculature, or inappropriate vascularisation of pathological tissue.
NUPR1 (+); nuclear protein upregulated.	A stress protein.
NFE2L2 (+); a nuclear factor that regulates the production of anti-oxidant proteins.	Tissue responding to oxidative stress by making more anti-oxidant defences.
MYC (+); a phosphoprotein involved in regulating cell cycle proteins and apoptosis proteins.	Altered cell turnover and tissue repair.
SOD2 (-) encodes superoxide dismutase	Loss of enzymatic anti-oxidant defence.
Genes altered because of adverse physiology/pathophysiology.	
TFRC (+); transferrin receptor protein, upregulated.	Management of iron homeostasis.
CCL5 (+); T-cell activation regulator increased.	Activation of the immune response in progress.
BCL2 (+); regulator of apoptosis increased.	Altered programmed cell death, attempts to maintain tissue health.
C1RPB (+); cold stress gene, upregulated.	Disturbances to thermoregulation.
APOE (+): apolipoprotein involved in fat metabolism, upregulated.	Implicated in neurological plaque formation and other fatty change pathology in the CNS.
XBP1 (-); encodes histocompatibility complex protein	Non-specific immune response decreased.

Table S3 Gene expression changes associated with the steps in the adverse outcome pathway (AOP) for Al_2O_3 in Alzheimer's disease.

**Genes and their products**	**Possible adverse outcome**
Direct to the nerve cell.	
GRIN1 (+); encodes part of the NMDA receptors in the hypothalamus and elsewhere.	Synthesis of new receptors as NMDA receptor functions are already impaired; such as long term potentiation and memory formation.
Indirect effects on glial cells and housekeeping of the CNS environment.	
CAT (-); encodes a subunit of catalase, downregulated.	Enzymatic anti-oxidant defence impaired
SFEBF1 (+); sterol regulatory element binding transcription factor.	Altered low density lipids and cholesterol. Lipid chemistry of plaque formation in the brain.
CASP7 (-) and CASP3 (+); caspase family of proteases involved in apoptosis.	Unclear, activation of one type of protease and not another.
BAX (-); factor that accelerates programmed cell death, downregulated.	Rate of normal cell death in tissue housekeeping unaltered.
STAR (-); encodes steridogenic acute regulatory protein. Controls cholesterol transfer inside mitochondria.	Cholesterol synthesis decreased, beneficial if plaques present, but potentially adverse to the insulation of nerves.
NFE2L2 (+); a factor that regulates genes involved in antioxidant defences.	Up regulation of anti-oxidant defence in response to oxidative stress.
FASN (+); encodes fatty acid synthase. Synthesis of long chain fatty acids, upregulated.	Fatty acids available to make plagues in the brain tissue.
RUNX2 (-) gene involved in synthesis of bone, teeth and cartiliage.	Appropriate down regulation, not wanted in the brain.
ABCA1 (-); ATP binding cassette transporter. Lipid and phospholipid flux through cell membranes, down regulated.	Reduce lipid availability, especially to apolipoprotein. Possibly beneficial with respect to slowing plaque formation.
Genes altered because of adverse physiology/pathophysiology.	
CXCR4 (+); chemokine receptor, upregulated; activates chemotaxis in lymphocytes.	Immune response initiated.
CD36 (-); encodes membrane protein involved in fatty acid transport. Role in inflammation and phagocytosis.	Inflammation reactions decreased (a step in immunosuppression).
AGT (+); encodes angiotensinogen.	Response to high blood pressure and/or salt.
JUNB (+); transcription factor involved in growth.	Inappropriate stimulation of growth process, possible pathology such as reactive hyperplasia and plaque formation.
TF (+); gene encoding transferrin protein, upregulated.	Iron homeostasis in the tissue.

Table S4 Gene expression changes associated with the steps in the adverse outcome pathway (AOP) for AgNPs in Amyotrophic lateral sclerosis (ALS).

Genes and their products	Possible adverse outcome
Direct to the nerve cell.	
Effects on ion channel genes and neurotransmitters not up or downregulated.	
Indirect effects on glial cells and housekeeping of the CNS environment.	
HMOX1 (-); encodes part of heme oxygenase, downregulated.	Catalytic degradation of damaged heme proteins is slowed (tissue repair).
ALDOA (+); encodes fructose-biphosphate aldolase protein.	Upregulation of glycolytic enzymes – possibly indicative of tissue hypoxia.
PSMB3 (+) and PSMC4 (+); genes encoding proteasome subunit protein upregulated.	Lysosomal activity increased; housekeeping of protein debris in the CNS.
Genes altered because of adverse physiology/pathophysiology.	
PRDX2 (+); encodes part of a peroxiredoxin. A family of antioxidant enzymes that reduce hydrogen peroxide.	Response to oxidative stress.
TRAF2 (+); tissue necrosis receptor associated factor.	Necrosis present in the tissue.
ITGAM (-); encodes integrin subunit M, downregulated.	Supressed immune functions such as less ability for agglutination reactions and phagocytosis of compliment-coated particles.
ERO1A (+); endoplasmic reticulum oxidoreductase	Response to oxidative stress

About INSIdEnano

INSIdE nano is a graphical tool that highlights connections between phenotypic entities based on their effects on the genes. The database behind the tool is a network whose nodes are grouped into four categories:

- Nanomaterials
- Drugs
- Chemicals
- Diseases

For each element, information regarding its effects on the genes is known. Edge weights in the network explain how similar is the effect, on the genes, of each couple of nodes.

Data

INSIdEnano integrates four different types of phenotypic entities:

- **Nanomaterials**: Gene expression data of different human cell types exposed to different ENMs coming from the NanoMiner (http://compbio.uta.fi/estools/nanommune/index.php) project. NanoMiner is a collection of 634 samples derived from human primary cells and cell lines exposed to ENMs.
- **Drugs**: Gene expression data for drug treatments was downloaded from the Connectivity Map (https://www.broadinstitute.org/cmap/) (CMap) web page
- **Diseases** and **Chemicals**: Manually curated information about chemical-gene and disease-gene interaction were retrieved from the Comparative Toxicogenomics Database (http://ctdbase.org/) (CTD) website

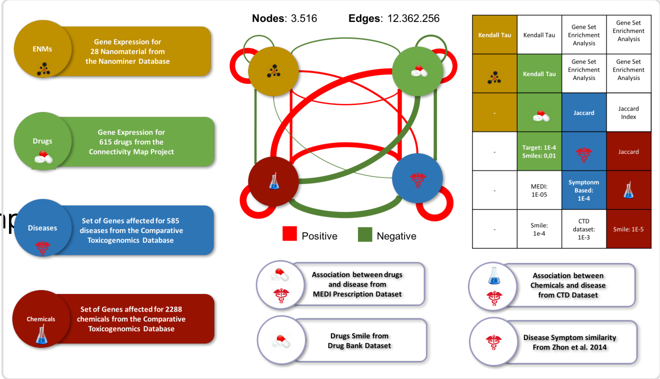

INSIdEnano data organization

For each phenotypic entity, a list of associated genes is given. In particular, a set of genes is associated to each disease and chemical, while an ordered list of genes resulting from differential expression analysis is built for each drug and ENM in the data set. In order to construct a network of similarity between the phenotypic entities, the pair-wise similarity between each possible pair of entities was evaluated. Because of the different nature of the information (sets or ordered list of genes), different measures were applied to evaluate the pair-wise similarity:

- The **Jaccard Index** was used to compile the similarity between sets of genes (e.g. two

diseases, two chemicals, or a disease and a chemical)
- The **Kendall Tau distance** was used to compute the similarity between ordered lists of genes (e.g. two nanomaterials, two drugs, or a nanomaterial and a drug)
- The **Gene Set Enrichment Analysis** was used to compute the similarity between sets and ordered lists of genes (e.g. a chemical/disease and a nanomaterial/drug)

The pairwise similarity matrix was used as an adjacency matrix to construct a weighted undirected network where the nodes are the entities and the similarity between them represents the weight of the edge. Each similarity measure has a different range of values both positive and negative. In order to make them comparable, these values were scaled into uniform range 0-1 by means of the cumulative function. Unlike the similarity value, the signs have not been altered; then edges in the network have a sign that indicates if the correlation between a couple of nodes is positive or negative.

Tool description and tutorials

Exploitative Analysis. The system provides two major functions for the exploitative analysis of the data set. The former is the visualization of the phenotypic network and the latter is the visualization clustering of the phenotypic entities.

Query Analysis. The tool provides two different types of queries. The former, called simple query, allows the user to investigate connections of a specific element in the network. Given a node and a threshold, the tool shows all its neighbours divided into four categories: nanomaterials, diseases, drugs and chemicals. For each phenotypic entity in the query output the tool highlights its position in the ranking of the neighbours and the information indicating whether the connection is already known in the literature or not. Moreover, the tool displays the connection distributions for the query input.

The latter, called conditional query analysis, allows the users to query the network by applying different filters. The user can specify more than one item for each data type, the level of similarity necessary to report a connection between two selected items, the number of items that must be in the same resulting cliques and the number of query items being connected to the other nodes in the sub-network. The tool gives two different outputs: firstly for each item, it creates a sub-network of all the elements connected to the input phenotypic entities with a connection stronger than the selected threshold; secondly it analyses the sub-networks and founds out all the patterns (cliques) of three or four different types of phenotypic entities each other significantly interconnected. The cliques are then clustered with respect to the nature of the connection between two items.

Network Browser Tutorial

The network browser tool allows the user to display and interact with the network. Due to the high

dimensionality of the network the tool allows the user to display only part of it.

How do I perform the simple query analysis?

Default Interface

1. Click on Browse in the navigation bar
2. The tool, by default, displays the subnetwork of all the elements associated to WCCo with threshold 1%
3. The network browse panel is available on the left side of the screen. It can be used to modify the network layout or filter out edges and nodes of the network
4. Network statistics are showed on the right part of the screen
5. Nodes can be clicked for more details

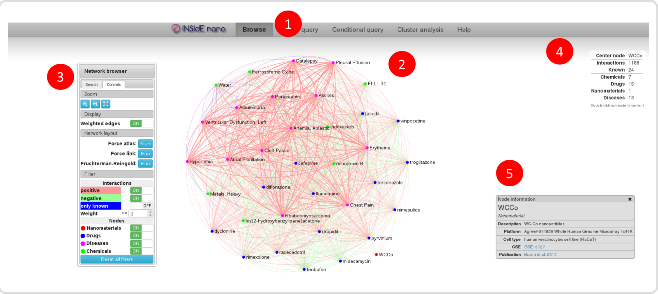

INSIdEnano network browser

Querying the network

In order to visualize the information related to a specific node the user can follow these steps:

1. Click on the search button in the network browser pane
2. Insert the name of the node to investigate (in the example TIO2T20)
3. Specify the percentage of top interaction to visualize
4. Press on the button "Update network" to visualize the result

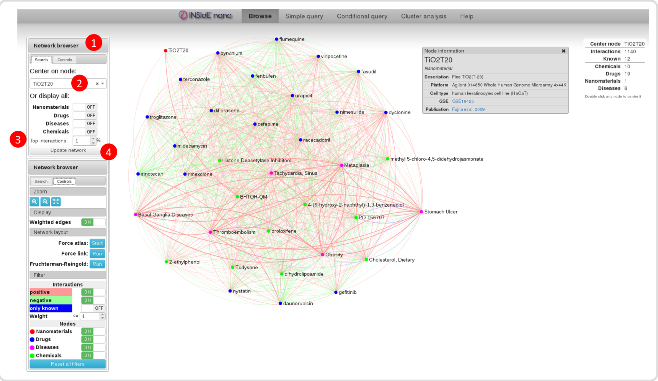

Performing a query within the network

Displaying complete subnetworks

The user can also display the subnetwork of a specific class of nodes for, example nanomaterials. In order to perform this operation, the user must follow the following steps:

1. Open the search tab in the network browser panel
2. Select the category of objects to display
3. Click on the "Update network" button

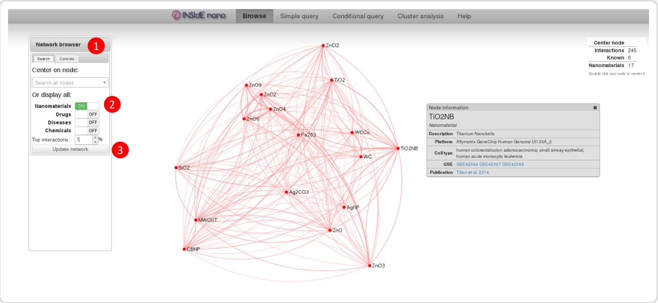

Subnetwork of all nanomaterials

Simple query tutorial

How do I perform the simple query analysis?

Input

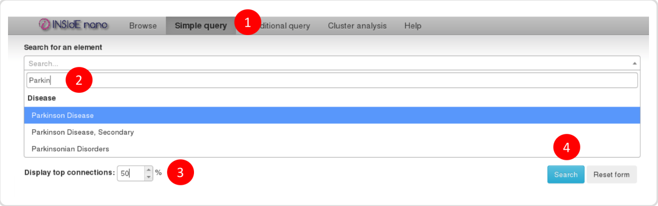

1. Click on Simple Query in the navigation bar
2. Choose the name of the element from the element list
3. Insert the connection strength threshold
4. Click on Search

Simple query form

Query Example: Parkinson Disease with threshold 50%

Results

Results will be displayed on three tabs:

1. The first one reports all the entities connected to the given element listed by category (nanos, drugs, chemicals and diseases)
2. The second one gives information about the connection distributions. We can see that Parkinson has 75% of positive and 25% of negative connections with nanomaterials in the following example.
3. The third one gives information about the distribution weights.

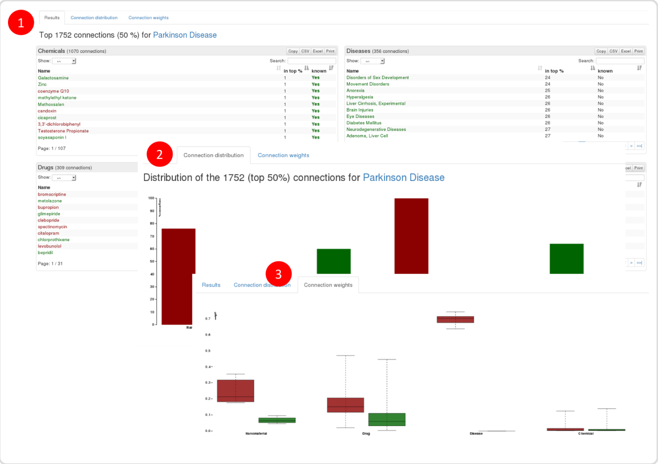

Simple query results

The user can investigate the result section by:

1. Sorting the elements by their position in the ranking or sort the elements based on the fact that the connection is known in literature or not. For example, sorting drugs by the known connection we find 'levodopa' to be connected to Parkinson with rank 9.
2. Clicking on the name of the element more information is available. The tool displays their ATC code, unique IDs and gives links to external resources in case of drugs.
3. Clicking on the levodopa Wikipedia link we discover that levodopa is used to increase dopamine concentrations in the treatment of Parkinson's disease
4. The colours of the element names give information about the positive (red) or negative (green) type of connection

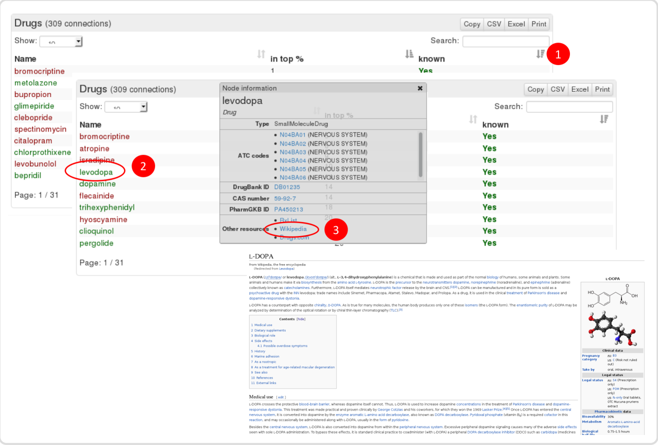

Simple query results

Conditional query tutorial

How do I perform the conditional query analysis?

Input

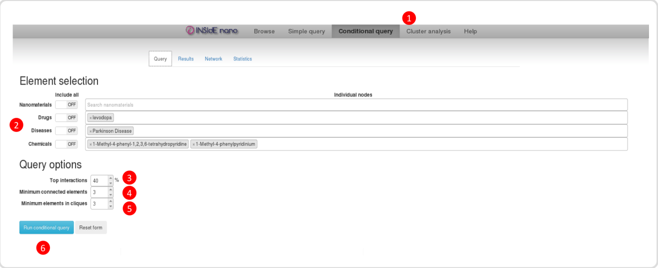

1. Click on Conditional Query in the navigation bar

2. Insert the subset of elements to investigate divided by the four categories: nanomaterials, drugs, diseases and chemicals.
   - N.B.: insert elements of at least two categories
   - Insert one, more than one, or all the element of a specific category
   - In order to insert all the element of a category, click on the "include all" button and switch it from OFF to ON:

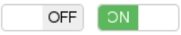

3. Insert the connection strength threshold
4. Insert the "**minimum connected elements**" threshold. This threshold allows to control the number of neighbours of the elements of interest to be selected. In particular, it indicates how many of the elements of interest are to be connected to a neighbour to consider it part of the analysis.
5. Insert the "**minimum elements in cliques**" threshold. This threshold allows to control the number of elements of interest to be in the final cliques.
6. Click on Run conditional query

Conditional query output

The output is displayed in three different tabs:

Clique List

The tab named "Results" contains the list of cliques. The panel is organized in the following manner:

1. The left panel shows the types of cliques. Cliques are categorized based on the classes of the objects that they contain. The user can choose the group to visualize by clicking on its button
2. The "Matching cliques" panel reports a table with the list of cliques.
3. Cliques can be filtered based on the fact that at least one of the interaction represented are already known in literature
4. Moreover, the list can be filtered based on the sign of the edge between two object of different classes
5. Elements can be searched in the table by typing their name in the search field
6. By clicking on a row representing the cliques another panel named clique information appear. It gives information regarding the kind of connection between the nodes.

Conditional query form

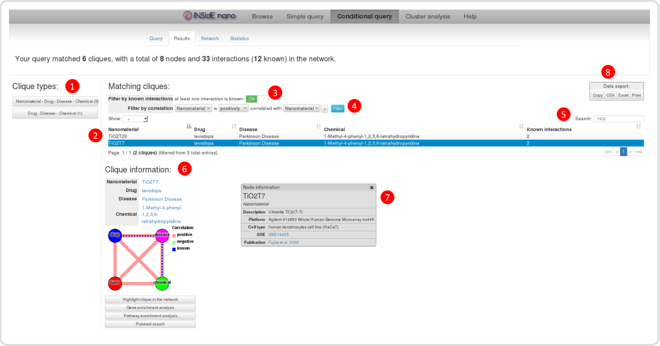

Conditional query results

7. In this tab each entity name is clickable in order to show its details
8. Tables can be exported by using the data export panel

Clique Investigation

Clique information panel has four buttons that can be used to further investigate the selected clique.

1. The first one highlights the clique in the subnetwork obtained from the analysis
2. The second one shows a table containing information about the common genes affected by all element in the clique. A green arrow means that the gene is upregulated by the element; a red arrow means it is downregulated by the element; two black arrows means that the genes is affected but we do not know if it is up or down regulated.
3. The third one shows the genes affected by each element of the clique, divided in groups. The groupings come from the gene sets collection of MSigDB. The Jaccard index has been evaluated for each set of the MSigDB database and the set of genes affected by each element. A threshold based on this index can be used for visualization purpose. Only sets of genes with Jaccard index higher than the one specified will be displayed. Each set name is expandable in order to inspect how the genes it contains behave with respect to the phenotypic entity.
4. This button allows to perform a PubMed search by using the elements in the clique. The default query is displayed. The user can either use the default query or change it before submitting to PubMed.

Subnetwork

This tab shows the subnetwork obtained from the conditional query analysis. The user can:

1. control the network from the control panel. It is divided into six main parts:
    a. zoom in and out the network
    b. network layout algorithms
    c. node size: the weighted size of the is the node degree.
    d. edge thickness: the weighted thickness is the strength of correlation between the connected nodes
    e. edge filter
    f. node filter

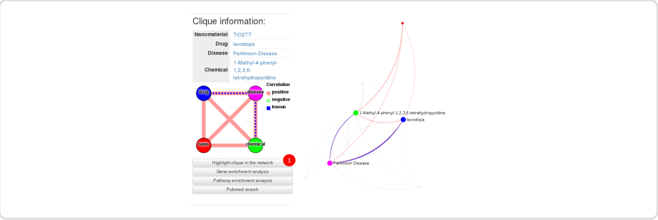

1. Highlighting a clique in the network

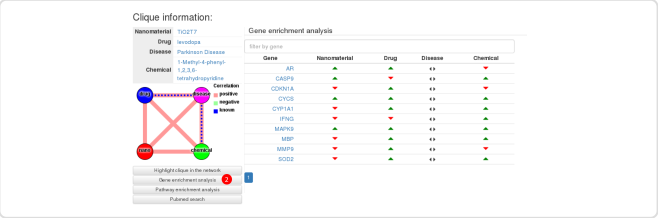

2. Gene enrichment information

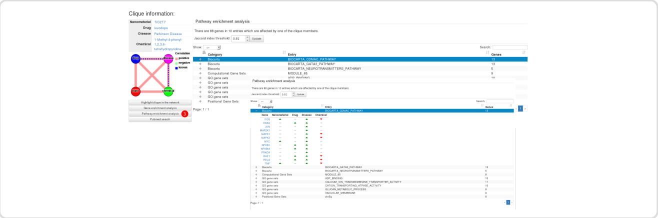

3. Pathway enrichment information

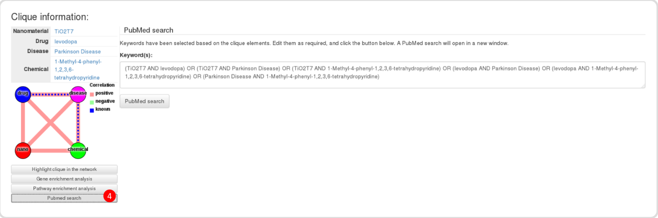

3. PubMed query interface

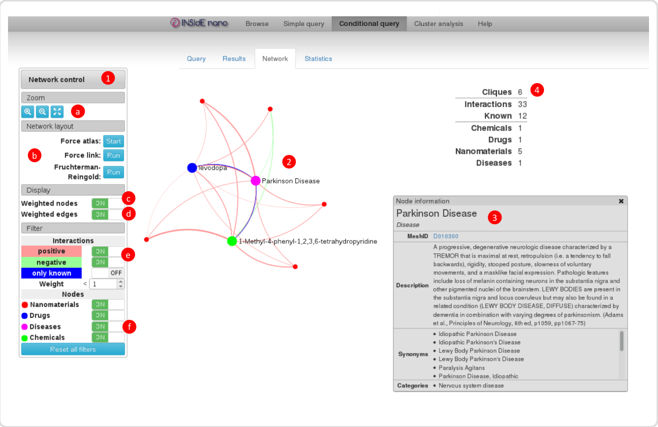

Conditional query subnetwork tab

2. visualize the network and interact with it. One can for example click and drag the nodes or display their names by hovering the mouse pointer
3. display detailed information about a node by clicking it
4. inspect the network statistics

Statistics

This tab allows the user to visualize various statistics about the resulting cliques. In particular, it counts how many time a couple of objects are connected in the cliques. The user needs to specify the two classes of objects to investigate and the tool returns a bubble plot where the bubbles radii indicate the number of times these two objects are connected.

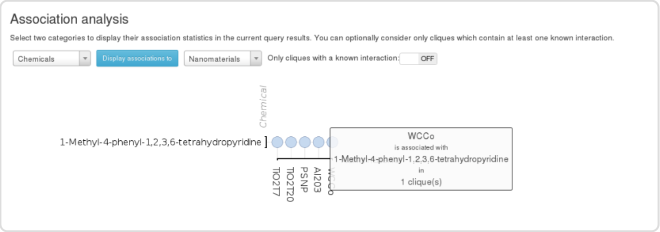

Conditional query results statistics

Clustering analysis tutorial

The Cluster Analysis panel gives the possibility to investigate how nanomaterials, drugs, disease and chemical are grouped each other and which genes are most important to each of them.

How do I perform the clustering analysis?

1. Click on Cluster Analysis in the navigation bar
2. Choose the kind of items to investigate, for example nanomaterials. The tool displays the list of all nanomaterials grouping based on their gene expression value
3. The user can click on a nanomaterial name to obtain more information in the pane on the right
4. The information panel contains information about the nanomaterials gene expression experiments. Moreover, the list of up and down regulated genes coming from the differentially expressed analysis is displayed
5. By clicking on the name of the genes its Gene card webpage is displayed.

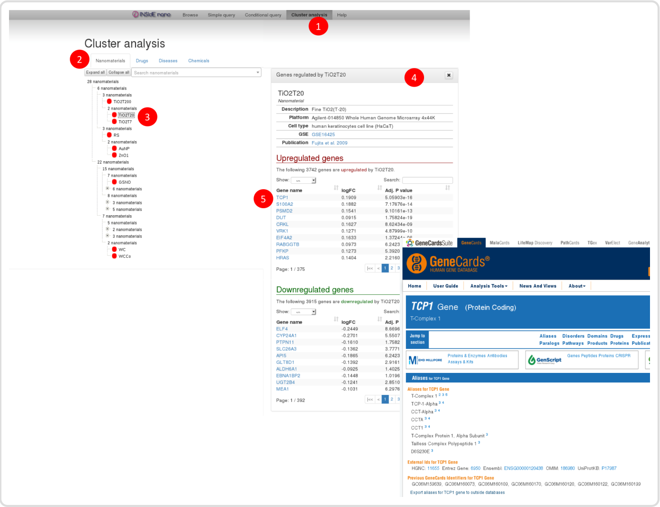

Clustering analysis

Support

The development of INSIdEnano was supported by the European Commission (FP7-NANOSOLUTIONS, Grant Agreement No. 309329).
